# A Water-Soluble Fluorogenic Probe to Quantify Azide Concentrations in Biologically Relevant Environments

**DOI:** 10.1021/acs.biomac.6c00253

**Published:** 2026-07-22

**Authors:** Lotte Gerrits, Lisa Verdellen, Marie Peeters, Arwen Gelderman, Ulysse J. Gross, Behrad Shaghaghi, Roel Hammink, Paul H. J. Kouwer

**Affiliations:** † Institute for Molecules and Materials, 6029 Radboud University, Heyendaalseweg 135, Nijmegen 6525 AJ, The Netherlands; ‡ Institute for Chemical Immunology, Geert Grooteplein 28, Nijmegen 6525 GA, The Netherlands; § Department of Medical BioSciences, Radboudumc, Geert Grooteplein 26, Nijmegen 6525 GA, The Netherlands; ∥ Division of Immunotherapy, Oncode Institute, Radboudumc, Nijmegen 6525 GA, The Netherlands

## Abstract

Fluorogenic probes are highly useful tools for the detection of target molecules in chemical biology and (bio)­material science, as they can often be applied in situ and do not depend on purification. Over the past years, a variety of alkyne-based fluorogenic probes have been proposed to detect azides, paving the way for live cell imaging and biomaterial analysis. Unfortunately, undesirable photophysical properties and poor solubility in aqueous solutions hamper the widespread use of these fluorogenic compounds. This work describes a water-soluble azide probe with high fluorescence enhancement (130-fold increase in intensity) upon reaction. We find accurate determination of azide densities on a polymer scaffold at low micromolar concentrations in PBS buffer. Additionally, by functionalizing the probe with biomolecules, we built a single-step (bio)­molecule labeling and analysis vehicle. As proof of principle, a peptide was conjugated to polymer scaffolds and conversions could be accurately followed in situ. Altogether, our probe is a promising new chemical biology tool for analysis in physiological conditions.

## Graphical abstract

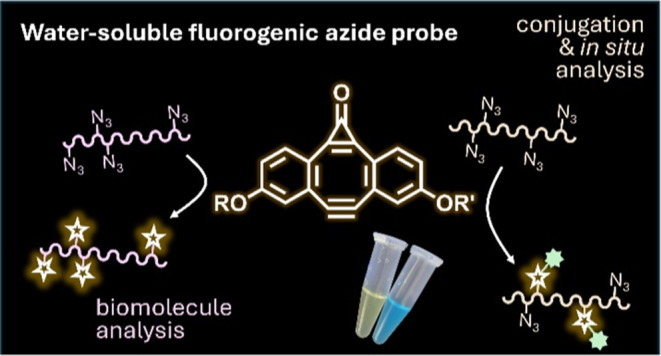

## Introduction

In fluorogenic compounds, fluorescence enhancement is observed after a reaction between the compound and target molecule occurs. Compared to traditional fluorescent dyes, these probes show much better signal-to-noise (S/N) ratios in fluorescence microscopy, especially in wash-free conditions. The superior S/N ratio makes them attractive for selective labeling strategies in chemical biology, particularly when the reaction is bioorthogonal. More specifically, the development of bioorthogonal fluorogenic probes has proven highly effective for applications such as live-cell imaging.^1–6^ Beyond chemical biology, the use of fluorogenic probes is extremely powerful in the analysis of (bio)­materials that often use bioorthogonal chemistry for postmodification strategies.^7^ They can be used to selectively detect functional groups, like azides, in biomaterials, which enables scientists to determine (bio)­molecule conjugation conversions without the need for laborious work-ups. Furthermore, employing fluorogenic reactions in aqueous solutions provides information about the biomaterial in a biologically relevant setting.

Various fluorogenic molecules that are based on the cycloaddition reaction between alkynes and azides have been developed. CalFluor azides were successfully used for in vivo imaging of glycans in zebrafish^8,9^ but require copper­(I), which hampers its widespread use. The strain-promoted azide–alkyne cycloaddition (SPAAC)-based probe CoumBARAC (Figure 1a) is copper-free, however, the triazole product suffers from a low quantum yield, from a modest increase in fluorescence intensity compared to the cyclooctyne, and from low wavelength UV light excitation.^10^ While another coumarin-fused cyclooctyne, CoumOCT (Figure 1a), exhibits improved quantum yields and has been successfully used for glycoconjugate imaging in live cells,^4^ its application remains limited,^11^ perhaps due to its laborious synthesis.

**Figure 1:**
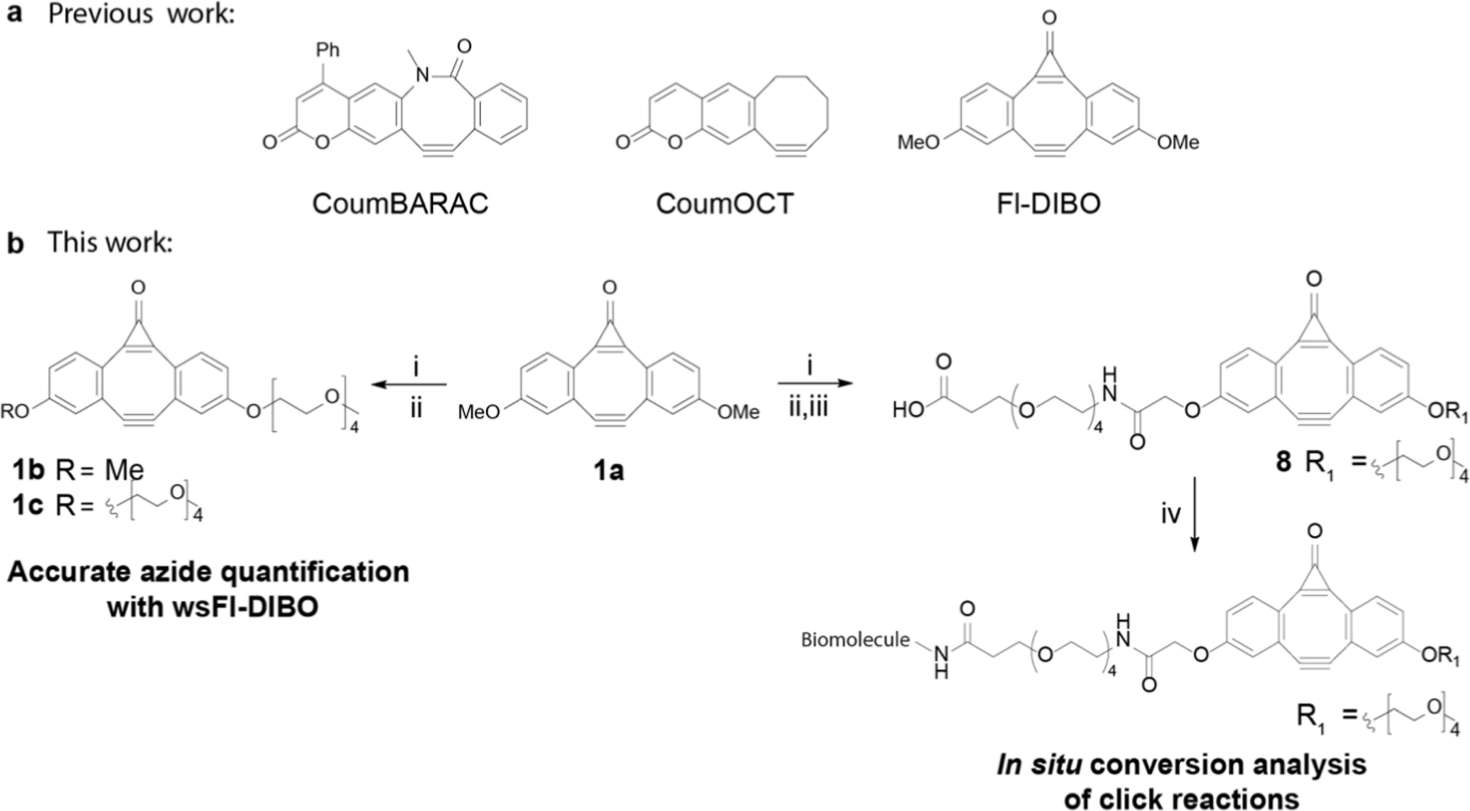
Overview of fluorogenic azide probes. (a) Previously reported fluorogenic compounds that react with azides via SPAAC reactions. (b) Synthesis of a water-soluble fluorogenic azide probe (wsFl-DIBO, 1c) (c) (i) BBr_3_, DCM, −78 °C to rt, 72 h, no workup; (ii) Ts-TEG-OMe, K_2_CO_3_, DMF, 50 °C, 18 h, 7.5% (**1b**) and 20% (**1c**). Synthesis of biomolecule functionalized wsFL-DIBO probe, (i) BBr_3_, DCM, −78 °C to rt, 72 h, no work up; (ii) 1. bromoacetamido-PEG_4_-*t*-butyl ester, K_2_CO_3_, DMF, 45 °C, 72 h 2. Ts-PEG_4_-OMe, K_2_CO_3_, DMF, 45 °C, 18 h, 11%; (iii) HCl in dioxane (4M), DCM, 0 °C to rt, 92 h, 71% (**8**); (iv) 1. *N*-hydroxysuccinimide, MES buffer, rt, 1 h, no work up; 2. NH_2_–Biomolecule, borate buffer 50 mM pH = 8.5, rt, 18 h, no work up.

Boons and co-workers developed Fl-DIBO (**1a**, Figure 1a), a cyclopropenone derivative of the Sondheimer diyne that forms a fluorescent triazole upon a SPAAC reaction with azides.^12^ The Fl-DIBO triazole shows desirable photophysical properties such as a large Stokes shift, a decent molar extinction coefficient, and an excitation wavelength above 350 nm. Despite its moderate fluorescence quantum yield,^12,13^ Fl-DIBO was successfully used to detect low concentrations of sodium azide in 1:1 mixtures of dioxane and HEPES buffer.^14^ Its poor water solubility, however, effectively blocks application of the probe in fully aqueous environments.

In this paper, we address the hydrophobic nature of the Fl-DIBO probe by incorporating oligo­(ethylene glycol) tails on a Fl-DIBO core (**1b**, **1c**, Figure 1b). The fluorescence intensity of the triazole products that are formed upon cycloaddition with an azide in PBS was greatly enhanced for these water-soluble Fl-DIBO derivatives in comparison to the original Fl-DIBO product. We demonstrate the potential of the water-soluble probes as a highly effective tool to detect azide concentrations in aqueous solutions at micromolar concentrations, for small molecules and for polymeric scaffolds. Additionally, we use an analogous approach to develop a functional Fl-DIBO probe that enables biomolecule attachment and analysis in a single-step (Figure 1b). In this way, the conversion of grafting reactions of biomolecules to scaffolds can be traced in situ, again at micromolar concentrations. In short, we expanded the fluorogenic probe toolbox with a water-soluble variant of Fl-DIBO that enables scientists to analyze materials in aqueous environments, compatible biomaterials.

## Results and Discussion

### Synthesis and Characterization of a Water-Soluble Fl-DIBO Azide Probe

The water-soluble fluorogenic azide probe (wsFl-DIBO) **1c** was prepared from Fl-DIBO **1a** (Figure 1b), that was synthesized via an optimized reported protocol (Scheme S1).^12^ Boron tribromide was used to cleave the methyl ethers of **1a**, after which a Williamson ether synthesis reaction with tosyl-functionalized tetra­(ethylene glycol) monomethyl ether produced wsFl-DIBO **1c** as the major product (20% yield over two steps). In addition, we isolated the monosubstituted tetra­(ethylene glycol) monomethyl ether Fl-DIBO (monoTEG-Fl-DIBO) **1b** as a side product (7.5% yield over two steps) due to incomplete ether cleavage in the previous step. The synthetic biomolecule conjugation route follows a similar strategy, vide infra.

The photophysical properties of both **1b** and **1c** upon SPAAC reaction with azido-PEG_3_-amine were compared to those of **1a** reacted under the same conditions (Figure 2a). To this end, **1a**, **1b** and **1c** (100 μM) were in PBS and an excess of azido-PEG_3_-amine was added. At this cyclooctyne concentration, **1a** did not fully dissolve in PBS and remained suspended even after multiple rounds of sonication while **1b** and **1c** gave clear solutions, indicating that the addition of ethylene glycol tails significantly improved the water solubility of the compounds. After 24 h of incubation, we characterized the photophysical properties of the corresponding triazoles, **2a**, **2b** and **2c**, and observed strong fluorogenic properties of **1c** upon reaction with azides with the naked eye (Figure 2b). The recorded excitation and emission spectra of **1c** and **2c** confirmed that the emitted fluorescence intensity increased 130-fold upon the click reaction (Figure 2c). Excitation of **1c** at 370 nm produced a weak emission band centered around 425 nm, with a low quantum yield of 1.4%, while the excitation of **2c** after triazole formation resulted in strong fluorescence with a maximum intensity at 475 nm and a quantum yield of 22.5% (Tables 1 and S1). Excitation of the triazole products of **2a** and **2b** did yield an enhancement in fluorescence in comparison with the unreacted compounds **1a** and **1b** (Figure S1), albeit much less pronounced than for **1c**. Additionally, the recorded quantum yields were also lower, 1.2% and 4.7% for **2a** and **2b**, respectively (Table 1), demonstrating the superior water-solubility of probe **1c**.

**Figure 2:**
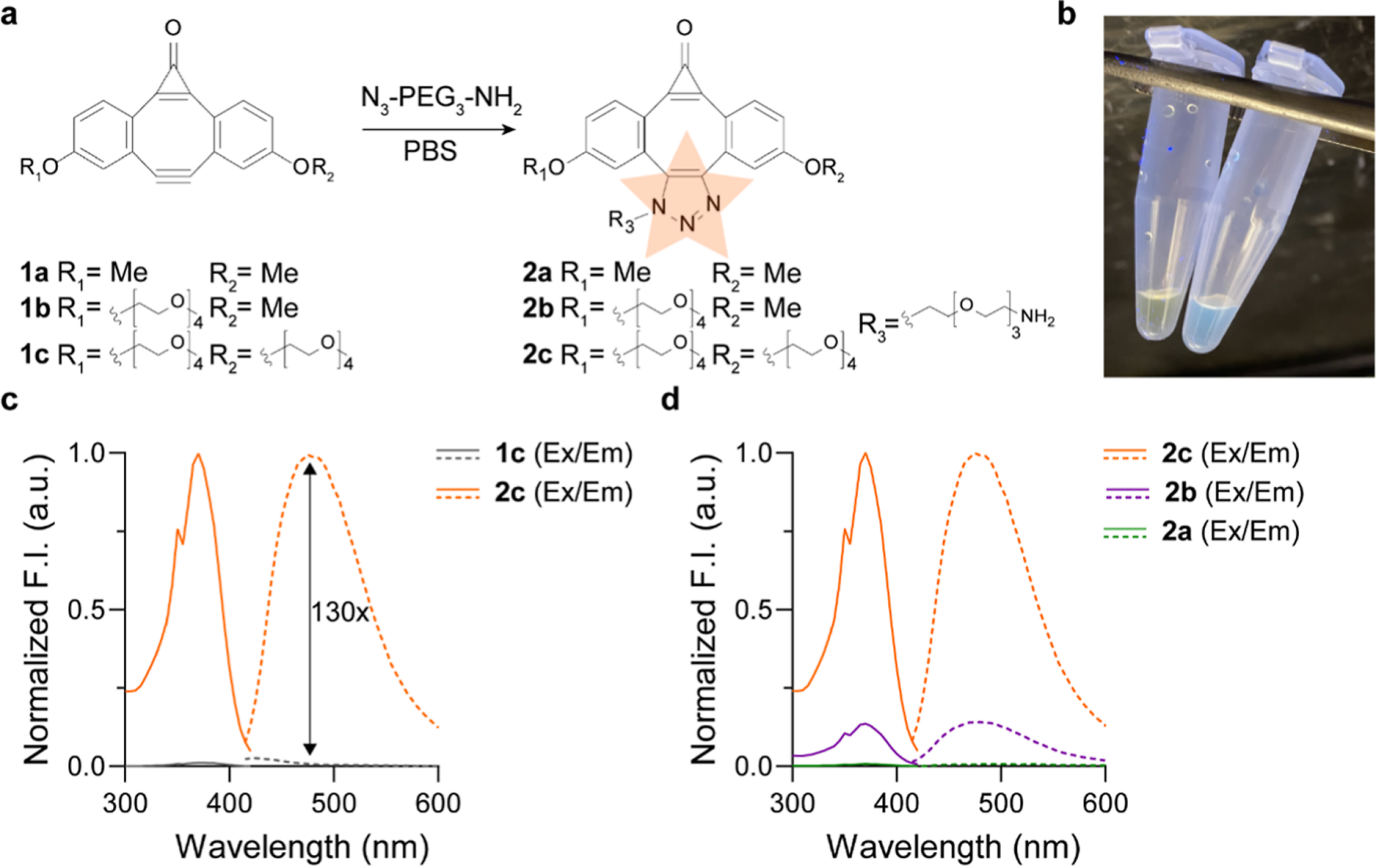
Enhanced fluorescence of wsFl-DIBO (**1c**) after triazole formation. (a) SPAAC reaction between the Fl-DIBO compounds (**1a**, **1b** and **1c**) and azide-PEG_3_-amine. (b) Inset picture to compare the visual fluorescence of unreacted wsFl-DIBO **1c** (left microtube) and the corresponding triazole **2c** (right microtube) under UV light (366 nm). (c) Excitation (solid lines) and emission (dashed lines) spectra of **1c** (gray) and the corresponding triazole **2c** (orange). (d) Excitation (solid lines) and emission (dashed lines) spectra of triazoles Fl-DIBO **2a** (green) **2b** (purple) and **2c** (orange) in PBS at 20 °C.

**Table 1:** Photophysical Properties of the Triazole Compounds **2c**, **2b** and **2a** in PBS at 20 °C

compound	λ_abs_ (nm)	λ_em_ (nm)	ε_abs_ (M^–1^ cm^–1^)^a^	stokes shift (cm^–1^)	*Q*_f_ ^b^
**2a**	366	495	219	7120	0.012
**2b**	362	480	300	6791	0.047
**2c**	360	475	1006	6725	0.225

a Determined at 366 nm for **2a**, 362 nm for **2b** and 360 nm for **2c**.

b Fluorescence quantum yield *Q*_f_ determined with quinine sulfate (1.0 M in H_2_SO_4_) as standard.

The difference in fluorescence intensity becomes obvious when comparing the excitation and emission spectra of **2a**, **2b** and **2c** (Figure 2d). Notably, **2c** shows a 152-fold increased fluorescence intensity in comparison to **2a**. We hypothesize that the observed decrease in fluorescence for **2a** and **2b** results from the reduced water solubility of the cyclooctynes **1a** and **1b** and their corresponding triazoles **2a** and **2b**. It is well-known that aggregation of fluorescent dyes can reduce the fluorescence intensity.^15,16^ Introduction of one tetra­(ethylene glycol) tail improves the solubility of **1b** in PBS, however, the lower fluorescence intensity of triazole **2b** compared to wsFl-DIBO triazole **2c** suggests that **2b** did not fully dissolve molecularly, but aggregated to some extent.

We further investigated the photophysical properties of the triazole compounds **2a**, **2b** and **2c** and found that all compounds possess large Stokes shifts of 7120, 6791, and 6725 cm^–1^, respectively (Table 1), which is desired as a small Stokes shift can lead to the reabsorption of the emitted photons.^17^ The apparent brightness, defined as the product of the quantum yield and the molar extinction coefficient,^18^ is 227-fold increased upon triazole formation for **1c**, while brightness only increased 3-fold for **1a** and 14-fold for **1b**. Based on our findings, we identified **1c** (wsFl-DIBO) as a suitable fluorogenic compound for reaction with azides in aqueous solutions.

### Proof-of-Concept a Posteriori Conversion Analysis of Click-Conjugation Reactions

To demonstrate the potential of wsFl-DIBO as a probe for the detection of azides in aqueous solutions, we used poly­(isocyano peptides)^19^ (PICs) as a model system. PICs are a unique class of oligo­(ethylene glycol)-grafted synthetic semiflexible polymers with a helical conformation,^20^ analogous to many biomacromolecules.^21^ Postfunctionalization of PIC with variety of (bio)­molecules, including proteins, peptides, antibodies, dyes and cross-linkers through SPAAC chemistry.^7,22^ Upon heating, aqueous solutions of PIC form gels.^23^ The gelation temperature *T*_gel_ is tuned by the length of the ethylene glycol chain, for tri­(ethylene glycol)­PIC (triPIC), *T*_gel_ ∼18 °C, and for tetra­(ethylene glycol)­PIC (tetraPIC), *T*_gel_ ∼40 °C.^24^ At 37 °C, TriPIC forms hydrogels with mechanical properties that closely mimic the native cell environment, which, after decoration with biomolecules find application as a synthetic cell culture matrix and in regenerative medicine.^25–29^ TetraPIC remain soluble at physiological temperatures and find application in immunotherapies after decoration with immunomodulatory molecules such as antibodies and cytokines.^30,31^

For both polymers, the postfunctionalization approach employs the SPAAC reaction of cyclooctyne-modified (bio)­molecules with the azide-modified PICs (Figure 3). The latter is obtained by copolymerization of azide-appended monomers, typically in a feed fraction of 0.033. Two outstanding challenges, however, are the quantitative analysis of the azide density in PIC-N_3_, as well as the conversion of the SPAAC reaction.^30^ PICs and an increasing body of azide-containing molecular building blocks including nano and microparticles, will strongly benefit from an straightforward analytical tool for in situ determination of micromolar azide concentrations without the need for workup.

**Figure 3:**
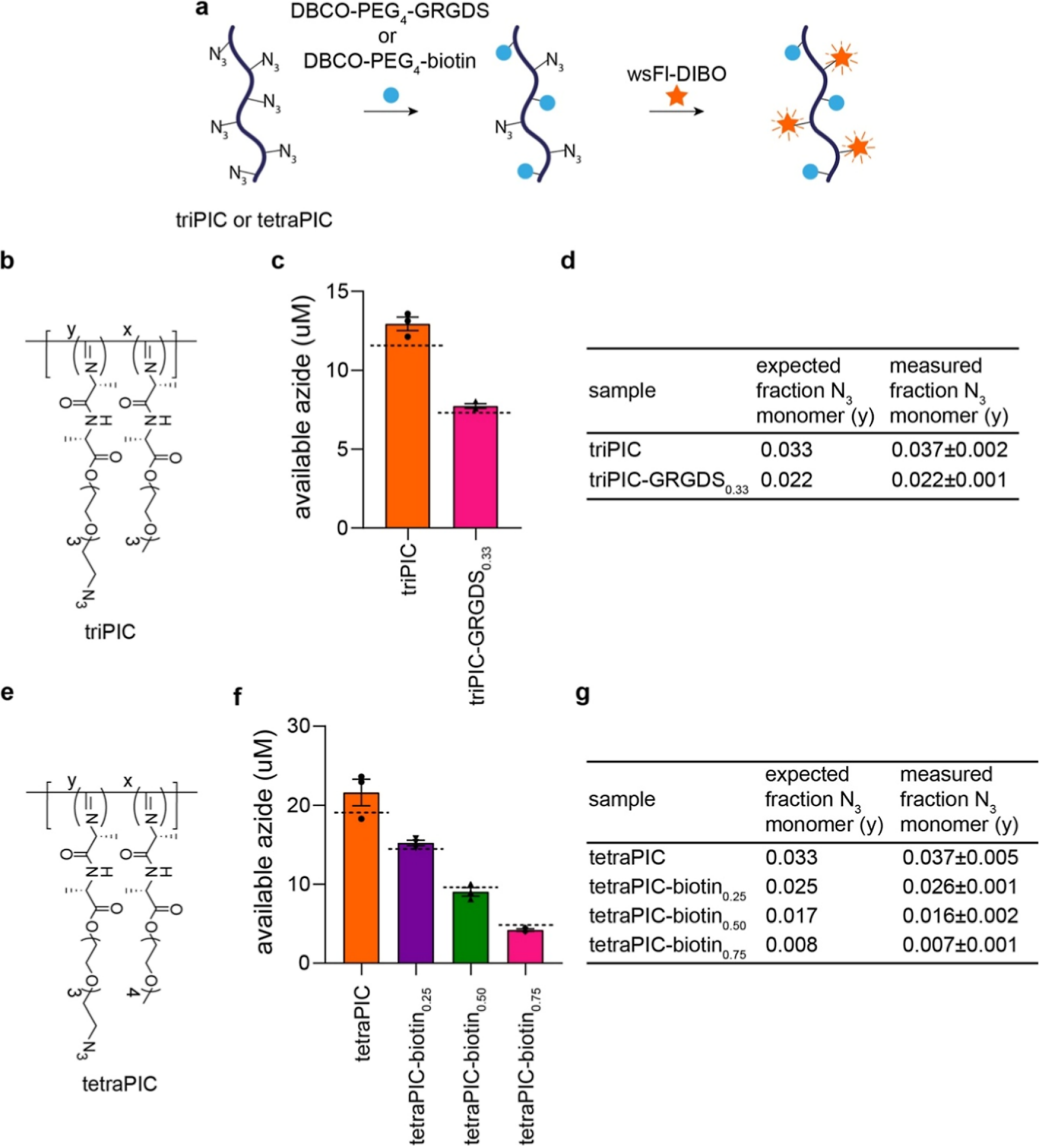
Azide quantification on triPIC and tetraPIC scaffolds. (a) Schematic overview of GRGDS or biotin conjugation to triPIC/tetraPIC and subsequent wsFl-DIBO conjugation for free azide concentration analysis on the scaffolds. (b) Chemical structure of triPIC scaffold. (c) Measured available azide concentrations for triPIC scaffolds before and after GRGDS conjugation. Dashed lines indicate the expected available azide concentration assuming a fraction of azide monomer (*y*) in triPIC is 0.033 (as in the monomer feed ratio during the polymerization) and assuming full conversion of the peptide conjugation reaction. Data are represented as mean ± SEM, *N* = 3. (d) Table showing the expected fraction of azide groups on the triPIC scaffolds and the measured fraction calculated from the wsFl-DIBO fluorescence experiment. Data are represented as the mean ± SD, *N* = 3. (e) Chemical structure of tetraPIC scaffold. (f) Measured available azide on concentrations for tetraPIC scaffolds before and after biotin conjugation. Dashed lines indicate the expected available azide concentration assuming a fraction of azide monomer (*y*) in tetraPIC is 0.033, assuming feed ratios and full conversion of biomolecule grafting. Data are represented as mean ± SEM, *N* = 3. (g) Table showing the expected fraction of azide groups on the triPIC scaffolds and the measured fraction calculated from the wsFl-DIBO fluorescence experiment. Data are represented as the mean ± SD, *N* = 3.

Addressing a real-life problem,^22^ we employed the wsFl-DIBO probe to measure the azide concentrations in the native triPIC polymer and tetraPIC polymer (Figure 3) and of the polymers that are partially substituted with biomolecules; for triPIC, the frequently used cell-adhesive peptide GRGDS to form triPIC-GRGDS^32,33^ and for tetraPIC a biotin linker. Excess of wsFl-DIBO was added^34^ and the fluorescence intensity was recorded after 72 h. The concentration of free azides on the polymer chains was determined with a calibration curve from triazoles formed after cycloaddition between azido-PEG_3_-amine and wsFl-DIBO under similar reaction conditions (Figure S2a,b). From the calibration curve, we also determined the Level of Detection (LOD) and Level of Quantification (LOQ) as measures for the lowest amount of azide in a sample that can be detected (but not necessarily quantified as an exact value) and the lowest amount of analyte in a sample that can be quantitatively determined with acceptable accuracy and precision, respectively.^35^ For wsFl-DIBO, we found LOD = 0.07 μM and LOQ = 0.22 μM.

TriPIC-GRGDS_0.33_ was synthesized by reacting triPIC with 0.33 equiv of DBCO-PEG_4_-GRGDS, which is expected to reduce the density of available azide groups on the polymer. Then, native triPIC (triPIC-GRGDS_0_) and triPIC-GRGDS_0.33_ were incubated with wsFl-DIBO (72 h, 5 °C to avoid gelation). The fluorescence intensity measurements were converted to concentration of azides that were available on the polymers. Note that all reactions were carried out in triplo to demonstrate the reproducibility of the triazole formation. For triPIC-GRGDS_0_, we found an azide concentration of 12.9 μM (expected 11.6 μM based on monomer ratios, dotted line in Figure 3c) and for triPIC-GRGDS_0.33_, we measured a concentration of 7.7 μM (expected 7.7 μM based on 100% conversion of GRGDS conjugation, dotted line). The values correspond to a fraction azide-appended monomer of 0.037 and 0.022 for triPIC-GRGDS_0_ and triPIC-GRGDS_0.33_, respectively (Figure 3d).

Analogously, the tetraPIC scaffold was reacted with DBCO-PEG_4_-biotin in DBCO/N_3_ ratios of 0.25, 0.5 and 0.75, yielding samples tetraPIC-biotin_0–0.75_. The polymers were incubated with the wsFl-DIBO probe in water at 20 °C (in triplo). Fluorescence intensity experiments yielded azide concentrations of 21.6, 15.3, 9.0, and 4.2 μM (expected 19.3, 14.5, 9.7, and 4.8 μM, based on monomer feed ratio and full DBCO-PEG_4_-biotin conversion, dotted line in Figure 3f). The experimental and calculated azide concentrations in the polymer show excellent agreement (Figure 3g). Beyond the three technical replicates in this experiment, we also tested coupling efficiencies on three independently synthesized tetraPIC-biotin batches, all prepared at the same intended DBCO/N_3_ ratios. Fluorescence analysis yielded good agreement between different polymer batches (Figure S3). Altogether, we demonstrated that we can accurately determine micromolar range azide concentrations on PIC scaffolds under various conditions using wsFl-DIBO as a fluorogenic probe.

### Proof-of-Concept In Situ Conversion Analysis of Click-Conjugation Reactions

In the previous section, we decorated an azide-rich scaffold and only after the reaction was completed, the conversion and thus the degree of labeling could be determined. Despite the good results, the accuracy of this approach is likely to decrease at low conversions. In an alternative approach, the biomolecule of interest is attached to the wsFl-DIBO probe, and the conversion can be determined in situ from the fluorescence intensity (Scheme S2).

To generate a monofunctional wsFl-DIBO equivalent, we followed a similar approach as described previously: we demethylated Fl-DIBO **1a**, after which we formed the asymmetrically substituted *t*-butyl ester protected wsFl-DIBO **7** through one-pot Williamson ether synthesis by sequential addition of bromoacetamido-PEG_4_-*t*-butyl ester and tosylated tetra­(ethylene glycol) monomethyl ether (**10**), respectively. Subsequent removal of the *t*-butyl protection group with HCl afforded the carboxylic acid functionalized wsFl-DIBO (COOH-wsFl-DIBO, **8**, Figure 4a). Before setting out to functionalize (bio)­molecules with our bifunctional wsFl-DIBO, we first confirmed that probe **8** retained its fluorogenic capacity. We observed a 284-fold increase in fluorescence for corresponding triazole **9**, that was formed by SPAAC mediated cycloaddition of **8** with azido-PEG_3_-amine (Figure S4). The photophysical properties of **8** and triazole **9** were assessed (Table S2), and we found quantum yields of 0.17% and 31.2% respectively.

**Figure 4:**
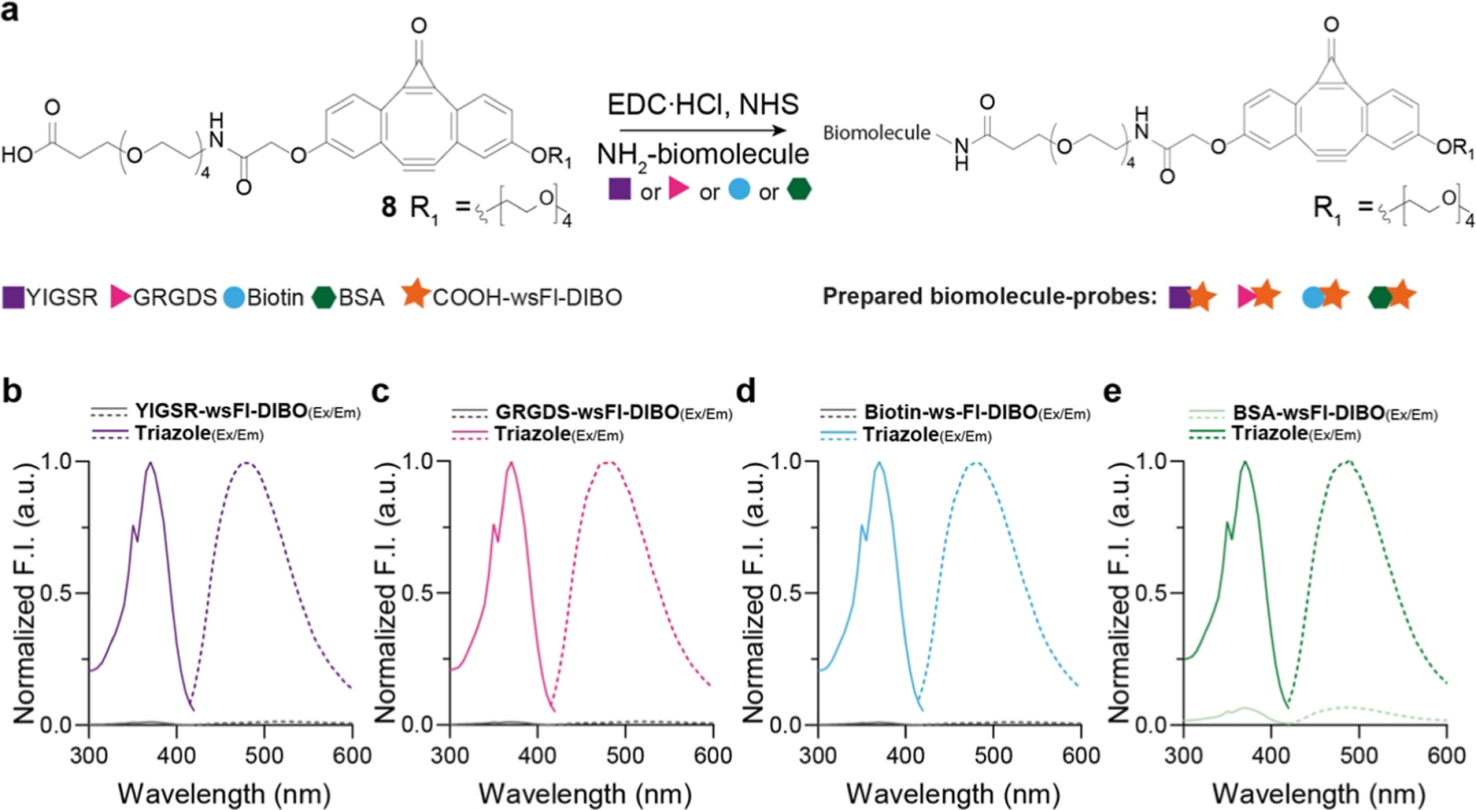
Bifunctional wsFl-DIBO. (a) Coupling of biomolecules (YIGSR, GRGDS, NH_2_–PEG_3_-biotin and BSA) to COOH-wsFl-DIBO through NHS-mediated chemistry. (b–e) Excitation (solid lines) and emission (dashed lines) spectra and corresponding triazole after incubation with N_3_–PEG_3_–NH_2_ in PBS at 20 °C of YIGSR-wsFl-DIBO (b), GRGDS-wsFl-DIBO (c), biotin-wsFl-DIBO (d) and BSA-wsFl-DIBO (e).

To demonstrate its versatility, we conjugated a variety of biomolecules to the carboxylic acid probe **8** using NHS mediated chemistry (Figure 4a). As a proof-of-principle, we used three biomolecules that are often conjugated to polymeric scaffolds: peptides (GRGDS and YIGSR) as well as amine-PEG_3_-biotin.^36–38^ Successful formation of GRGDS-wsFl-DIBO (**11**), YIGSR-wsFl-DIBO (**12**), and biotin-wsFl-DIBO (**13**) was confirmed using MALDI-TOF (Figures S5–S7). To establish that also larger biomolecules such as proteins can be incorporated, we conjugated the NHS-activated probe **8** to BSA. UV–vis spectroscopy after purification confirms the coupling (Figure S8) and the formation of BSA-wsFl-DIBO (**14**).

We then incubated the probe-functionalized biomolecules **11**–**14** with azide-PEG_3_-amine to assess their fluorogenic properties (Figures 4b–e and S9). Upon triazole formation, fluorescence significantly increased for all biomolecule-wsFl-DIBO compounds, confirming that the fluorogenic properties of wsFl-DIBO are retained after conjugation to biomolecules (Figure 4b–e). Altogether, these results show that our bifunctional wsFl-DIBO probe can be used for labeling of (bio)­molecules while retaining its unique properties.

Finally, we show that through the fluorogenic properties, the conjugation reaction can be followed in situ, also at low concentrations. To demonstrate, we conjugated the often-used GRGDS peptide, using GRGDS-wsFl-DIBO (**11**), to both triPIC and tetraPIC (Figure 5a). We studied two cases that we often use in the lab, a ‘high density’ polymer with the aim to convert all azides (uses 1 equiv of GRGDS) and a lower density polymer that uses 0.33 equiv of GRGDS and where two-thirds of the azides are available to conjugate other biomolecules. The reactions were carried out in PBS at 4 °C (triPIC, Figure 5b,c) or 20 °C (tetraPIC, Figure 5b,d) and the fluorescence intensity was measured as a function of reaction time. The solids line are fits to a second order reaction rate equation where both data sets for tetraPIC (and separately for TriPIC) are fitted together with a single set of parameters.

**Figure 5:**
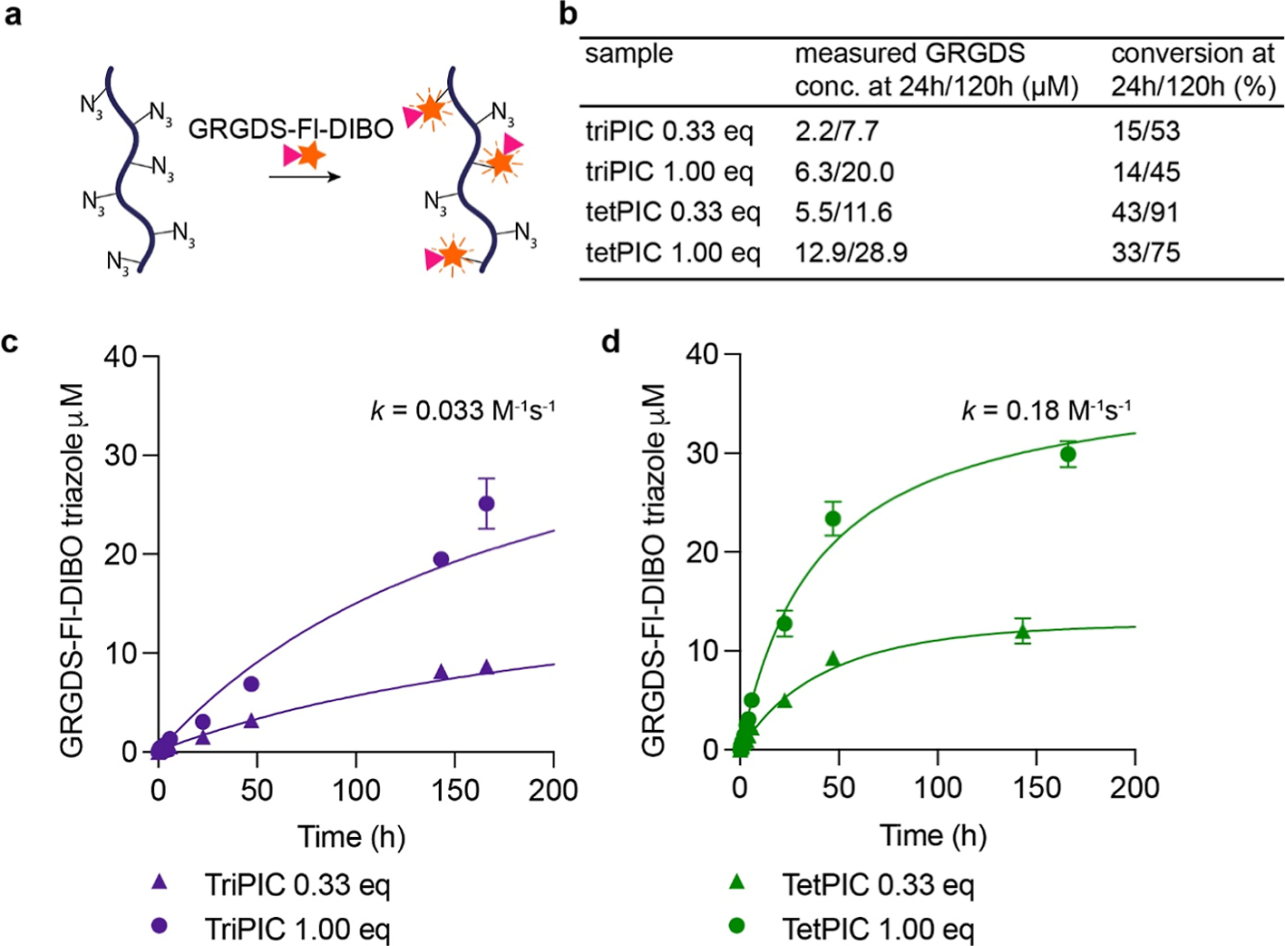
GRGDS-wsFl-DIBO coupling to PIC scaffolds. (a) Schematic overview of GRGDS-wsFl-DIBO conjugation to PIC scaffolds. (b) Determination of GRGDS-wsFl-DIBO concentrations and conversions on tri- and tetraPIC scaffolds after incubation with 0.33 or 1.0 equiv of GRGDS-wsFl-DIBO for 24 h or 120 h. Data is represented as the mean, *N* = 2 (with three technical replicates of each measurement). (c) Kinetic plot of the concentration of GRGDS-wsFl-DIBO triazole formed on triPIC scaffolds incubated with 0.33 or 1.0 equiv of GRGDS-wsFl-DIBO over time. Data are represented as mean ± SEM, *N* = 2 (with three technical replicates of each measurement). (d) Kinetic plot of the concentration of GRGDS-wsFl-DIBO triazole formed on tetraPIC scaffolds incubated with 0.33 or 1.0 equiv of GRGDS-wsFl-DIBO over time. Data are represented as mean ± SEM, *N* = 2 (with three technical replicates of each measurement). The solid lines in panels c and d are a fit to a second-order rate equation with second-order rate constant *k* as the sole fitting parameter for both data sets in each panel.

Clearly the reactions at 20 °C are faster than those at 4 °C. In the typical 10 s of micromolar concentration range, conversions after 24 h are still modest but clearly measurable (Figure 5b); driving the reaction to complete conversion takes more time or requires a further increase of temperature or an excess peptide. Although one should keep in mind that our reaction conditions are different, the observed rate constants largely match those of SPAAC reactions of other cyclooctynes.^39^ Overall, we conclude that incubation of GRGDS-wsFl-DIBO with PIC scaffolds results in good conjugation yields and eliminates the need for workup or additional steps for analysis.

## Conclusions

In this work, we developed a water-soluble fluorogenic probe, wsFl-DIBO, for the detection of azides in biologically relevant environments. While other strategies have been published,^40^ the advantage of fluorogenic dyes is that they can be used at low concentrations and without purification of the analyte.

We based the design of our probe on Fl-DIBO, a fluorogenic compound that cannot be used in biological systems due to its hydrophobicity, yet exhibits suitable photophysical properties.^12,18^ Introduction of ethylene glycol tails yielded wsFl-DIBO, which showed excellent solubility in aqueous solutions. SPAAC-mediated cycloaddition with a water-soluble aliphatic azide demonstrated the fluorogenic ability of wsFl-DIBO: the emission of the triazole product was enhances >100-fold compared to the emission of the cyclooctyne. Furthermore, we found that the triazole product exhibits a high quantum yield (Φ_f_ = 0.225), a good molar extinction coefficient (ε = 1006 M^–1^ cm^–1^), and a large Stokes shift (6725 cm^–1^), which are desirable photophysical properties for the detection of azides in biological milieus.^41^

As proof-of-concept, the new fluorogenic dye was used to determine the azide concentration in polymer scaffolds that are commonly used in our laboratory. We find that azide densities can be reliably detected into the single digit micromolar concentration regime, which makes wsFl-DIBO a great analytical tool for many applications that use SPAAC conjugation strategies. Potentially, the application of our wsFl-DIBO probe could be expanded to the quantitative detection of other functional groups that are often applied in conjugation reactions, such as diazo-derivatives, nitrones, nitrile oxides, and sydnones. Earlier studies have reported that the fluorescence of the parent compound, Fl-DIBO, is also enhanced upon reaction with these chemical handles.^42,43^

One step further, probe modification allows in situ analysis of the bioconjugation reaction, at the same concentration levels. The incubation temperature significantly influences the reaction rate of the cycloaddition between the probe and the azide-containing scaffolds. Conjugation of other biomolecules to our probe derivate will further establish its potential as a bifunctional linker, for example, antibody- or cytokine functionalized Fl-DIBO constructs could significantly reduce the laborious preparation and analysis required in the development of biomaterials for the stimulation of T cells.^30,31,44,45^ Based on our findings we conclude that the bifunctional wsFl-DIBO has great potential as a novel tool for biomaterial development.

## Materials and Methods

### Organic Synthesis of Water-Soluble Azide Probes

#### 1,2-Bis­(3-methoxyphenyl)­ethyne (**3**)^12^

*N*,*N*-Diisopropylethylamine (1.56 mL, 9.36 mmol) was added dropwise to a solution of 3-ethynylanisole (0.480 mL, 3.84 mmol), 3-iodoanisole (0.360 mL, 3.01 mmol), tetrakis­(triphenylphosphine)­palladium(0) (204 mg, 0.18 mmol) and copper­(I) iodide (66.0 mg, 0.330 mmol) in THF (15.0 mL). The reaction mixture was then refluxed for 18 h. The reaction mixture was concentrated in vacuo and purified by FCC (SiO_2_, EtOAc/Heptane, 1:8, v/v), after which recrystallization in heptane afforded **3** (716 mg, 3.00 mmol, 99%) as a white solid. ^**1**^ **H NMR** (400 MHz, CDCl_3_) δ: 7.28 (t, *J* = 7.9 Hz, 2H, Ar–H), 7.16 (dt, *J* = 7.6, 1.2 Hz, 2H, Ar–H), 7.10 (dd, *J* = 2.7, 1.4 Hz, 2H, Ar–H), 6.93 (ddd, *J* = 8.3, 2.6, 1.0 Hz, 2H, Ar–H), 3.86 (s, 6H, CH_3_).^**13**^ **C NMR** (101 MHz, CDCl_3_) δ: 159.37 (COCH3), 129.43 (Ar–C), 124.23 (Ar–CH), 124.19 (Ar–C), 116.37 (Ar–CH), 115.03 (Ar–CH), 89.13 (C≡C), 55.32 (CH3). **LCMS** (*m*/*z*): [M + H]^+^ calcd for C_16_H_15_O_2_^+^, 239.1; found, 238.8.

#### (Z)-1,2-Bis­(3-methoxyphenyl)­ethene (**4**)^12^

A solution of **3** (666 mg, 2.82 mmol), Lindlar’s catalyst (135 mg, 20% w/w), and quinoline (0.720 mL, 6.10 mmol) in heptane (17.0 mL) were stirred at rt under H_2_ flow for 45 min. The mixture was filtered over Celite, washed with EtOAc (20.0 mL), and concentrated in vacuo. The product was isolated by FCC (SiO_2_, EtOAc/Heptane, 1:8, v/v) to afford **4** (665 mg, 2.77 mmol, 99%) as a colorless oil. ^**1**^ **H NMR** (400 MHz, CDCl_3_) δ: 7.14 (t, *J* = 7.9 Hz, 2H, Ar–H), 6.85 (dt, *J* = 7.6, 1.2 Hz, 2H, Ar–H), 6.80 (dd, *J* = 2.6, 1.6 Hz, 2H, Ar–H), 6.74 (ddd, *J* = 8.3, 2.7, 0.9 Hz, 2H, Ar–H), 6.57 (s, 2H, C = CH), 3.66 (s, 6H, CH_3_). ^**13**^ **C NMR** (101 MHz, CDCl_3_) δ: 159.37 (COCH_3_), 138.54 (CCH = CH), 130.35 (CH = CH), 129.19 (Ar–CH), 121.52 (Ar–CH), 113.81 (Ar–CH), 113.31 (Ar–CH), 55.06 (CH_3_). LCMS (*m*/*z*): [M + H]^+^ calcd for **LCMS** (*m*/*z*): [M + H]^+^ calcd for C_16_H_17_O_2_^+^, 241.1; found, 241.8.

#### (Z)-4,9-Dimethoxy-1*H*-dibenzo­[a,e]­cyclopropa­[*c*]­cyclooctene-1-one (**5**)^12^

A solution of aluminum chloride (247 mg, 1.85 mmol) and tetrachlorocyclopropene (0.110 mL, 1.84 mmol) in DCM (23 mL) was stirred under Ar flow at rt for 30 min and cooled to −78 °C. A solution of **4** (356 mg, 1.50 mmol) in DCM (8.50 mL) was added and the resulting mixture was stirred at −78 °C for 1.5 h, followed by addition of aluminum chloride (240 mg, 1.80 mmol) and additional stirring for 1 h. The reaction was then allowed to warm to rt. After stirring for 2 h, H_2_O (30 mL) was added, and the reaction mixture was stirred for 30 min. The crude product was then extracted with DCM (3 × 16.0 mL), dried over MgSO_4_, concentrated in vacuo and purified by FCC (SiO_2_, MeOH in DCM, 0 → 2%, v/v) to obtain **5** (205.2 mg, 0.707 mmol, 47%) as a yellow solid. ^**1**^ **H NMR** (400 MHz, CDCl_3_) δ: 7.44 (d, *J* = 8.4 Hz, 2H, Ar–H), 6.69 (dd, *J* = 8.4, 2.6 Hz, 2H, Ar–H), 6.60 (d, *J* = 2.6 Hz, 2H, dd, *J* = 2.6, 1.6 Hz, 2H, Ar–H), 5.99 (s, 2H, CH = CH), 3.82 (s, 6H, CH_3_). ^**13**^ **C NMR** (101 MHz, CDCl_3_) δ: 163.39 (Ar–C), 152.28 (C=O), 147.56 (Ar–C), 139.88 (Ar–C), 136.01 (Ar–CH), 131.73 (CH = CH), 121.62 (Ar–CH), 115.19, 113.32 (Ar–CH), 55.52 (CH_3_). **LCMS** (*m*/*z*): [M + H]^+^ calcd for C_19_H_15_O_3_^+^, 291.1; found, 291.6.

#### 6,7-Dibromo-4,9-dimethoxy-6,7-dihydro-1*H*-dibenzo­[a,e]­cyclopropa­[c] Cycloocten-1-one (**6**)^12^

Bromine (3.25 mL, 0.264 M in DCM) was added dropwise to a cooled (0 °C) solution of **5** (137.1 mg, 0.472 mmol) in DCM (7.70 mL). Subsequently, the mixture was allowed to warm to rt, stirred for 4 h, and was quenched with sodium thiosulfate (sat. aq., 10 mL). The aqueous layer was extracted with DCM (3 × 15.0 mL) and the combined organic layers were dried over MgSO_4_, filtered, and concentrated in vacuo. The crude product was isolated by FCC (SiO_2_, MeOH in DCM, 0 → 2%, v/v) to afford **6** (137.5 mg, 0.305 mmol, 65%) as a colorless solid. ^**1**^ **H NMR** (400 MHz, CDCl_3_) δ: 8.07 (d, *J* = 8.4 Hz, 2H, Ar–H), 7.06 (dd, *J* = 8.5, 2.5 Hz, 2H, Ar–H), 6.98 (d, *J* = 2.6 Hz, 2H, Ar–H), 5.76 (s, 2H, CHBr), 3.93 (s, 6H, CH_3_). ^**13**^ **C NMR** (101 MHz, CDCl_3_) δ: 162.60 (Ar–C), 152.56 (C=O), 142.82 (Ar–C), 141.77 (Ar–C), 137.05 (Ar–CH), 118.15 (Ar–CH), 115.71 (Ar–C), 114.04 (Ar–CH), 55.76 (CH_3_), 50.36 (CH–Br). **LCMS** (*m*/*z*): [M + H]^+^ calcd for C_19_H_15_Br_2_O_3_^+^, 448.9; found, 449.0.

#### 4,9-Dimethoxy-6,7-didehydro-1*H*-dibenzo­[a,e]­cyclopropa­[c]­cycloocten-1-one (**1a**)^12^

A solution of potassium hydroxide (187 mg, 3.33 mmol) in EtOH (4.0 mL) was added to a solution of **5** (150 mg, 0.333 mmol) in ethanol (30.0 mL). The reaction mixture was stirred at rt for 18 h and quenched with an aqueous solution of HCl (1N, until pH ≤ 7). The mixture was then extracted with DCM (3 × 10.0 mL), and the combined organic layers were washed with Brine (sat. aq. 50.0 mL) and concentrated under reduced pressure. The residue was purified by FCC (SiO_2_, Acetone in DCM, 0–20%, v/v) affording pure cyclooctyne **1a** (58.1 mg, 0.202 mmol, 61%) as a yellow solid. ^**1**^ **H NMR** (400 MHz, CDCl_3_) δ: 7.47 (d, *J* = 8.5 Hz, 2H, Ar–H), 6.63 (dd, *J* = 8.5, 2.6 Hz, 2H, Ar–H), 6.45 (d, *J* = 2.6 Hz, 2H, Ar–H), 3.81 (s, 6H, CH_3_). ^**13**^ **C NMR** (101 MHz, CDCl_3_) δ: 163.24 (Ar–C), 136.61 (Ar–CH), 126.72 (Ar–C), 125.28 (Ar–C), 114.64 (Ar–CH), 113.55 (Ar–CH), 106.67 (Ar–C), 55.67 (CH_3_). **LCMS** (*m*/*z*): [M + H]^+^ calcd for C_19_H_13_O_3_^+^, 289.1; found, 289.3.

#### monoTEG-Fl-DIBO (**1b**) and wsFl-DIBO (**1c**)

Compound **1b** and **1c** were prepared in two steps via adaptation of earlier described protocols.^46,47^ First, a solution of **1a** (45.0 mg, 0.156 mmol) in dry DCM (17 mL) was cooled to −78 °C and stirred for 10 min under Ar flow before dropwise addition of Boron tribromide (2.81 mL, 1 M in DCM). The reaction mixture was stirred for 30 min at −78 °C, allowed to warm to rt and stirred for 72 h. Subsequently, the reaction mixture was quenched with ice-cold MeOH (16.0 mL) and concentrated over Ar flow. The crude intermediate was used in the next step without further workup. MeO-PEG_4_-OTs (**11**, 143 mg, 0.449 mmol) was added to a solution of the intermediate (23.0 mg, 0.089 mmol) in dry DMF (3.0 mL). Potassium carbonate (126.8 mg, 0.917 mmol) was added portion-wise until the reaction mixture reached pH ≥ 8. Subsequently, the reaction mixture was heated to 45 °C and stirred for 18 h. The mixture was then concentrated in vacuo and purified by FCC (SiO_2_, DCM/EtOAc, 9:1, v/v), which afforded **1b** (3.1 mg, 0.016 mmol, 7.5%) as a yellow oil and **1c** (11.3 mg, 0.017 mmol, 20%) as a yellow oil. **1b**: ^**1**^ **H NMR** (400 MHz, CDCl_3_) δ: 7.48 (dd, *J* = 8.5, 6.4 Hz, 2H, AR-H), 6.65 (ddd, *J* = 8.5, 4.7, 2.6 Hz, 2H, Ar–H), 6.48 (dd, *J* = 11.7, 2.6 Hz, 2H, Ar–H), 4.17–4.10 (m, 2H, CH_2_), 3.88–3.83 (m, 2H, CH_2_), 3.82 (s, 3H, CH_3_), 3.76–3.65 (m, 10H, CH_2_), 3.59–3.54 (m, 2H, CH_2_), 3.40 (s, 3H, CH_3_). ^**13**^ **C NMR** (101 MHz, CDCl_3_) δ: 163.24 (Ar–C), 162.50 (Ar–C), 153.62 (C=O), 146.44 (Ar–C), 146.35 (Ar–C), 136.61 (Ar–CH), 136.54 (Ar–CH), 126.73 (Ar–C), 126.68 (Ar–C), 125.37 (Ar–C), 125.26 (Ar–C), 115.12 (Ar–CH), 114.63 (Ar–CH), 114.22 (Ar–CH), 113.54 (Ar–CH), 106.70 (C≡C), 106.65 (C≡C), 71.95 (CH_2_), 70.91 (CH_2_), 70.64 (CH_2_), 70.62 (CH_2_), 70.54 (CH_2_), 69.35 (CH_2_), 67.90 (CH_2_), 59.06 (CH_3_), 55.67 (CH_3_). **LCMS** (*m*/*z*): [M + H]^+^ calcd for C_27_H_28_O_7_, 465.521; found, 465.328. **1c**: ^**1**^ **H NMR** (400 MHz, CDCl_3_) δ: 7.45 (d, *J* = 8.5 Hz, 2H, Ar–H), 6.64 (dd, *J* = 8.5, 2.6 Hz, 2H, Ar–H), 6.47 (d, *J* = 2.6 Hz, 2H, Ar–H), 4.15–4.08 (m, 4H, CH_2_), 3.87–3.78 (m, 4H, CH_2_), 3.74–3.61 (m, 20H, CH_2_), 3.58–3.51 (m, 4H, CH_2_), 3.38 (s, 6H, CH_3_). ^**13**^ **C NMR** (101 MHz, CDCl_3_) δ: 162.50 (Ar–C), 153.61 (C=O), 146.42 (Ar–C), 136.55 (Ar–CH), 126.69 (Ar–C), 125.36 (Ar–C), 115.12 (Ar–CH), 114.21 (Ar–CH), 106.68 (C≡C), 71.95 (CH_2_), 70.91 (CH_2_), 70.64 (CH_2_), 70.62 (CH_2_), 70.54­(CH_2_), 69.34­(CH_2_), 67.89­(CH_2_), 59.06­(CH_3_). **LCMS** (*m*/*z*): [M + H]^+^ calcd for C_35_H_45_O_11_^+^, 641.3; found, 641.7.

#### *t*-Butyl Ester-wsFl-DIBO (**7**)

Compound **7** was prepared through a similar method as **1b** and **1c**. First, a solution of **1a** (40.0 mg, 0.139 mmol) in dry DCM (15.0 mL) was cooled to −78 °C and stirred for 10 min under N_2_ flow before dropwise addition of Boron tribromide (2.20 mL, 1 M in DCM). The reaction mixture was stirred for 30 min at −78 °C, allowed to warm to rt and stirred for 72 h. Subsequently, the reaction mixture was quenched with ice-cold MeOH (16.0 mL) and concentrated over air flow. The crude intermediate was used in the next step without further workup. The crude product was dissolved in dry DMF (6.0 mL) and potassium carbonate (1.27 g, 9.20 mmol) was added portion-wise until the reaction mixture reached pH ≥ 8. A solution of bromoacetamido-PEG_4_-*t*-butyl ester (29.5 mg, 0.07 mmol) in dry DMF (1.50 mL) was added dropwise and the reaction was heated to 45 °C and stirred under N_2_ flow for 72 h. After full conversion of the starting material was confirmed via LCMS, a solution of MeO-PEG_4_-OTs (**10**, 231.8 mg, 0.642 mmol) in dry DMF (2.00 mL) was added, followed by addition of potassium carbonate (296 mg, 2.14 mmol). Subsequently, the reaction mixture was stirred for 96 h. The mixture was then concentrated in vacuo and purified by FCC (Al_2_O_3_, Acetone in DCM, 0 → 100%, v/v), which afforded **7** (12.8 mg, 0.016 mmol, 11%) as a yellow solid. ^**1**^ **H NMR** (400 MHz, CDCl_3_) δ: 7.47 (t, *J* = 8.4 Hz, 2H, Ar–H), 7.00 (t, *J* = 5.5 Hz, 1H, NH), 6.66 (ddd, *J* = 8.6, 6.0, 2.6 Hz, 2H, Ar–H), 6.49 (t, *J* = 3.0 Hz, 2H, Ar–H), 4.48 (s, 2H, CH_2_), 4.12 (dd, *J* = 5.7, 3.8 Hz, 2H, CH_2_), 3.84 (dd, *J* = 5.7, 3.7 Hz, 2H, CH_2_), 3.74–3.50 (m, 32H, CH_2_), 3.38 (s, 3H, CH_3_), 2.50 (t, *J* = 6.6 Hz, 2H, CH_2_), 1.44 (s, 9H, CH_3_). ^**13**^ **C NMR** (101 MHz, CDCl_3_) δ: 170.85 (C=O), 166.84 (C=O), 162.64 (Ar–C), 160.64 (Ar–C), 153.46 (C=O), 147.43 (C=C), 146.03 (C=C), 136.71­(Ar–CH), 136.49 (Ar–CH), 127.07 (Ar–C), 126.53 (Ar–C), 126.49 (Ar–C), 125.19 (Ar–C), 115.25 (Ar–CH), 114.82 (Ar–CH), 114.62 (Ar–CH), 114.31 (Ar–CH), 107.19 (C≡C), 106.19 (C≡C), 80.53­(*C*CH_3_), 71.93 (CH_2_), 70.88 (CH_2_), 70.61 (CH_2_), 70.57 (CH_2_), 70.52 (CH_2_), 70.34 (CH_2_), 70.30 (CH_2_), 69.59 (CH_2_), 69.30 (CH_2_), 67.92 (CH_2_), 67.30 (CH_2_), 66.88 (CH_2_), 59.03 (CH_3_), 38.92 (CH_2_), 36.21 (CH_2_), 28.08 (CH_3_). **LCMS** (*m*/*z*): [M + H]^+^ calcd for C_43_H_58_NO_14_^+^, 812.4; found, 812.4.

#### COOH-wsFl-DIBO (**8**)

Compound **7** (12.8 mg, 0.016 mmol) was dissolved in DCM (5.0 mL) and cooled to 0 °C. A solution of HCl in dioxane (0.243 mL, 4M) was added dropwise and the reaction mixture was allowed to warm to rt and stirred for 92 h. The reaction mixture was quenched with *t*-BuOH (2.00 mL), concentrated in vacuo and purified by FCC (SiO_2_, MeOH in DCM, 0 → 8% + Acetic Acid 2%, v/v), to afford **8** (8.60 mg, 0.011 mmol, 71%) as a yellow solid. **LCMS** (*m*/*z*): [M + H]^+^ calcd for C_39_H_50_NO_14_^+^, 756.3; found, 756.0 (Figure S10).

#### *p*-Toluenesulfonate Tetra­(ethylene Glycol) Monomethyl Ether (**10**)

A solution of NaOH (1.30 g, 32.6 mmol) in H_2_O (6.00) was added dropwise to a cooled (0 °C) solution of tetra­(ethylene glycol) monomethyl ether (5.02 g, 24.1 mmol) in THF (5.0 mL). The resulting mixture was vigorously stirred for 5 min followed by dropwise addition of a solution of *p*-toluenesulfonyl chloride (4.55 g, 23.9 mmol) in THF (35.0 mL). The reaction mixture was allowed to warm to rt and was stirred for 2 h. Cooled (0 °C) H_2_O (100 mL) was added, and the mixture was extracted with DCM (5 × 75.0 mL). The combined organic layers were dried over Na_2_SO_4_, filtered, and concentrated in vacuo. The crude product was isolated by FCC (SiO_2_, EtOAc) affording **10** (7.99 g, 22.0 mmol, 92%) as a clear oil. ^**1**^ **H NMR** (400 MHz, CDCl_3_) δ: 7.81 (d, *J* = 8.3 Hz, 2H, Ar–H), 7.35 (d, *J* = 7.8 Hz, 2H, Ar–H), 4.17 (t, *J* = 4.8 Hz, 2H, CH_2_), 3.74–3.52 (m, 14H, CH_2_), 3.39 (s, 3H, CH_3_), 2.46 (s, 3H, CH_3_). ^**13**^ **C NMR** (101 MHz, CDCl_3_) δ: 144.79 (Ar–C), 133.03 (Ar–C), 129.82 (Ar–C), 127.99 (Ar–C), 71.94 (CH_2_), 70.75 (CH_2_), 70.61 (CH_2_), 70.59 (CH_2_), 70.53 (CH_2_), 70.52 (CH_2_), 69.2 (CH_2_), 68.68 (CH_2_), 60.39 (CH_3_), 59.03 (CH_3_). **LCMS** (*m*/*z*): [M + Na]^+^ calcd for C_16_H_26_O_7_SNa^+^, 385.1; found, 385.1.

#### Biomolecule-wsFl-DIBO (**11**, **12**, **13**)

YIGSR-wsFl-DIBO (**11**), GRGDS-wsFl-DIBO (**12**), and Biotin-wsFl-DIBO (**13**) were prepared via a general protocol. EDC·HCl (0.149 mL, 0.034 M in MES buffer pH 5.5) and *N*-hydroxysuccinimide (0.149 mL, 0.034 M in MES buffer, pH 5.5) were added to a solution of **8** (1.9 mg, 0.0025 mmol) in MES buffer (0.302 mL, pH 5.5). The resulting mixture was incubated on a rotator at rt for 1 h. Then, the NHS-activated probe mixture (0.113 mL) was added to a solution of GYIGSR-peptide (for **11**, GeneCust, 0.089 mL, 5.91 mM in Borate buffer 50 mM pH = 8.5), GRGDS peptide (for **12**, Bachem, 0.089 mL, 5.91 mM in Borate buffer 50 mM pH = 8.5), or amine-PEG_3_-biotin (for **13**, Click Chemistry Tools, 0.089 mL, 5.91 mM in Borate buffer 50 mM pH = 8.5). Borate buffer was added (0.049 mL) and the resulting mixture was incubated on the rotator at rt for 18 h. MALDI-TOF indicated the successful formation of **11**, **12**, and **13**. All biomolecule-probes were directly used without further workup. **MALDI-TOF** (*m*/*z*): **11**, [M + H]^+^ calcd for C_67_H_92_N_10_O_22_, 1390.537; found, 1390.398. **12**, [M + H]^+^ calcd for C_56_H_77_N_9_O_22_, 1229.277; found, 1129.700. **13**, [M + Na]^+^ calcd for C_57_H_81_N_4_O_18_S, 1179.350; found, 1179.638 (Figures S5–S7).

#### BSA-wsFl-DIBO (**14**)

BSA-wsFl-DIBO (**14**), was prepared via a similar protocol as described above. In short, EDC·HCl (5.00 μL, 80.0 mM in MES buffer, pH 5.5) and *N*-hydroxysuccinimide (5.00 μL, 80.0 mM in MES buffer) were added to a solution of **8** (10.0 μL, 20.0 mM in MES buffer, pH 5.5). The resulting mixture was incubated on a rotator at rt for 1 h. The NHS-activated probe mixture (12.0 μL) was added to a solution of BSA (Sigma-Aldrich, 122 μL, 0.25 mM in Borate buffer 50 mM pH = 8.5) and the resulting mixture was incubated on the rotator at rt for 18 h. BSA-wsFl-DIBO (**14**) was purified using an Amicon centrifugal filter (10 kDaA MWCO, Merck). The degree of labeling (DOL) of the BSA with wsFl-DIBO was determined by measuring absorbance and fluorescence of **14** after incubation with an azide small molecule to afford the fluorescent triazole product. In short, **14** (42.0 μL, 0.23 mM) was incubated with azido-PEG_3_-amine (3.95 μL, 10 mM in DMSO) and incubated on a rotator at rt for 24 h. A calibration curve was made through reaction of COOH-wsFl-DIBO (**8**, 12.6 μL, 10 mM in DMSO) with azido-PEG_3_-amine (25.2 μL, 10 mM in DMSO) in PBS (382 μL) at rt for 24 h, followed by dilution in PBS to obtain samples with concentrations from 300 to 2.344 μM. Fluorescent intensities were measured (Tecan Spark M10, λ_ex/em_ = 370/475 nm) and a calibration curve was fitted using a 4PL curve fit. From the fluorescent intensities, the concentration of wsFl-DIBO on **14** was calculated. The measured absorbance afforded the BSA concentration of **14**. Taken together, the DOL of wsFl-DIBO calculated to be 2.5. Reactions were carried out in duplo. PBS and unfunctionalized BSA were used as controls.

### Absorption, Excitation, and Emission Spectra of the Water-Soluble Azide Probes

Absorption, excitation, and emission spectra of the unreacted probes (**1a**, **1b**, **1c**, **8**) and the corresponding triazoles (**2a**, **2b**, **2c**, **9**) after reaction with azido-PEG_3_-amine were recorded on a Tecan Spark M10 plate reader. In short, solutions of **1a**, **1b**, **1c** and **8** (4.5 μL, 10 mM in DMSO) in PBS (436.5 μL) were incubated with azido-PEG_3_-amine (9 μL, 10 mM in DMSO) on a rotator at rt for 24 h. In addition, solutions of **1a**, **1b**, **1c** and **8** (4.5 μL, 10 mM in DMSO) in PBS (445.5 μL) were subjected to incubation on a rotator at rt for 24 h as well. Excitation (300–420 nm), emission (420–600 nm) and absorbance (250–700 nm) spectra were recorded (Figures S1, S4 and S11). Reactions were carried out in duplo. PBS was taken along as control. The absorption wavelength (λ_abs_), emission wavelength (λ_em_), and Stokes shift for **1a**, **1b**, **1c**, and **8** and the corresponding triazole compounds (**2a**, **2b**, **2c**, **9**) were determined from the absorption, excitation, and emission spectra, respectively.

### Absorption, Excitation, and Emission Spectra of the Biomolecule-wsFl-DIBO Probes

Absorption, excitation, and emission spectra of the unreacted probes (**11**, **12**, **13**, **14**) and the corresponding triazoles after reaction with azido-PEG_3_-amine were recorded on a Tecan Spark M10 plate reader. In short, solutions of **11**, **12**, **13** and **14** (20 μL, 1.9 mM in Borate buffer) in PBS (392.8 μL) were incubated with azido-PEG_3_-amine (7.6 μL, 10 mM in DMSO) on a rotator at rt for 24 h. In addition, solutions of **11**, **12**, **13** and **14** (20 μL, 1.9 mM in Borate buffer) in PBS (400 μL) were subjected to incubation on a rotator at rt for 24 h as well. Excitation (300–420 nm), emission (420–600 nm) and absorbance (250–700 nm) spectra were recorded (Figures 4b–e and S9). Reactions were carried out in duplo. PBS was taken along as control.

### Determination of Quantum Yields of the Water-Soluble Azide Probes

Quantum yields for the triazole compounds (**2a**, **2b**, **2c**, **9**) were determined using the slope of the integrated fluorescence emission versus absorbance quinine sulfate in 0.1 M H_2_SO_4_ as a reference (Φ_f_ = 0.54 ± 0.03)^48^ In short, solutions of **1a**, **1b**, **1c** and **8** (15, 75, 75, and 20 μL, respectively, all 10 mM in DMSO) in PBS (final volume 600 μL, or 500 μL for **8** and **9**) were incubated with azido-PEG_3_-amine (7.50, 37.5, 37.5, and 10.0 μL, respectively, all 100 mM in PBS) on a rotator at rt for 72 h. In addition, solutions of **1c** and **8** (15, 75, 75, and 20 μL, respectively, all 10 mM in DMSO) in PBS (final volume 600 μL, or 500 μL for **8**) were subjected to incubation on a rotator at rt for 72 h as well. For all compounds, the absorbance at the respective λ_abs_ was recorded. When *A* ≥ 0.3, compound samples were diluted with PBS until *A* < 0.3. Dilutions series of **1c**, **8** and the triazole compounds (**2a**, **2b**, **2c**, **9**) were made, and absorbances and fluorescent intensities (λ_ex/em_ = 370/475 nm) were measured (Tecan spark M10). For each compound, eight data points were acquired with absorbances between 0.01 and 0.1 (100 μL per well). Reactions were carried out in duplo. DMSO in PBS and PBS were taken along as controls.

### Determination of Extinction Coefficients of the Water-Soluble Azide Probes

Extinction coefficients for the triazole compounds (**2a**, **2b**, **2c**, **9**) were determined on a Tecan Spark M10 plate reader, using 96-well plates and a path length of l = 0.294 cm. In short, **1a**, **1b**, **1c** and **8** (15, 75, 75, and 20 μL, respectively, all 10 mM in DMSO) were added to solutions of azido-PEG_3_-amine (7.50, 37.5, 37.5, and 10.0 μL, respectively, all 100 mM in PBS) in PBS (final volume 600 μL, or 500 μL for **8** and **9**) and incubated on a rotator at rt for 72 h. In addition, solutions of **1c** and **8** (15 and 20 μL, respectively, all 10 mM in DMSO) in PBS (final volume 600 μL, or 500 μL for **8**) were subjected to incubation on a rotator at rt for 72 h as well. Absorbances were recorded for all compounds. When *A* ≥ 0.3, samples of the compounds were diluted with PBS until *A* < 0.3. Dilutions series of **1c**, **8** and the triazole compounds (**2a**, **2b**, **2c**, **9**) were made. Reactions were carried out in duplo. DMSO in PBS was used as a control.

### Determination of Azide Concentration on tetraPIC Scaffolds

tetraPIC scaffolds (**batch 1**, **2** and **3**) were prepared following an earlier reported protocol.^49^ DBCO-PEG_4_-biotin (0.00, 1.25, 2.50, or 3.75 μL, 10 mM in DMSO) was added to solutions of tetraPIC in PBS (300 μL, 2.00 mg/mL in PBS). The reaction mixtures were incubated on a rotator at rt for 3 h. Then, wsFl-DIBO (**1c**, 4.2 μL, 10 mM in DMSO) was added to the resulting Biotin-tetraPIC compounds (0.100 mg, 50.0, 50.2, 50.5, and 50.6 μL, respectively) and PBS was added to reach a final volume of 240 μL for each sample. The reaction mixtures were incubated on a rotator at rt for 72 h. A calibration curve was made through reaction of wsFl-DIBO (**1c**, 15.8 μL, 10 mM in DMSO) with azido-PEG_3_-amine (3.15 μL, 10 mM in DMSO) in PBS (431 μL) at rt for 72 h, after which the reaction mixture was diluted with PBS (431 μL), followed by further dilution in PBS to obtain samples with concentrations from 35.0 to 0.547 μM. The tetraPIC samples were diluted to final concentrations of 0.21 mg/mL PIC before measuring. Fluorescent intensities from the tetraPIC samples and calibration curve samples were measured (Tecan Spark M10, λ_ex/em_ = 370/475 nm). The calibration curve was fitted using a 4PL curve fit. Reactions were carried out in triplo. PBS was used as a control.

### Determination of Azide Concentration on triPIC Scaffolds

triPIC scaffolds (triPIC and triPIC-GRGDS) were prepared following an earlier reported protocol.^32^ wsFl-DIBO (**1a**, 1.4 μL, 10 mM in DMSO) was added to a solution of triPIC and triPIC-GRGDS (30 μL, 1 mg/mL) in PBS. The reaction mixtures were incubated on a rotator at 5 °C for 72 h and subsequently diluted with PBS (209 μL). A calibration curve was made through reaction of wsFl-DIBO (**1c**, 10.7 μL, 10 mM in DMSO) with azido-PEG_3_-amine (2.14 μL, 10 mM in DMSO) in PBS (431 μL) at 4 °C for 72 h, followed by dilution in PBS to obtain samples with concentrations from 47.6 to 0.372 μM. Fluorescent intensities from the triPIC samples and calibration curve samples were measured (Tecan Spark M10, λ_ex/em_ = 370/475 nm). The calibration curve was fitted using a 4PL curve fit. Reactions were carried out in triplo. PBS was used as a control.

### GRGDS-wsFl-DIBO Coupling to triPIC and tetraPIC Scaffolds

GRGDS-wsFl-DIBO (**12**, 7.1 μL (for 0.33 equiv) or 21.2 μL (for 1.00 equiv), 1.8 mM) was added to solutions of triPIC (209 μL, 2 mg/mL) in PBS (1000 μL final volume). The triPIC reaction mixtures were incubated on a rotator at 4 °C for 143 h. Similarly, GRGDS-wsFl-DIBO (**12**, 7.1 μL (for 0.33 equiv) or 21.2 μL (for 1.00 equiv), 1.8 mM) was added to solutions of tetraPIC (209 μL, 2 mg/mL) in PBS (1000 μL final volume). The tetraPIC reaction mixtures were incubated on a rotator at rt for 143 h. All PIC samples were diluted 4 times before measuring the fluorescence. A calibration curve was made through the reaction of COOH-wsFl-DIBO (**8**, 7.0 μL, 10 mM in DMSO) with azido-PEG_3_-amine (1.4 μL, 10 mM in DMSO) in PBS (191.6 μL) at rt for 47 h (70 μM probe), followed by dilution in PBS to obtain samples with concentrations from 17.5 to 0.137 μM. Fluorescent intensities from the triPIC and tetraPIC samples (at time points of 0.6 h, 1.15 h, 2.13 h, 3.25 h, 4.3 h, 5.9 h, 22.5 h, 47 h and 143 h) and calibration curve samples (at 47 h) were measured (Tecan Spark M10, λ_ex/em_ = 370/475 nm). The calibration curve was fitted using a 4PL curve fit. Reactions were carried out in duplo, with 3 technical replicates. PBS was used as a control. Conversions were fitted to a standard second order rate equation with known starting concentrations and the rate constant as only free fitting parameter. The two data sets of triPIC were fitted together in a single iteration, with the starting concentrations of triPIC the same (azide concentration [N_3_] = 44 μM) and those of **12** different for the two data sets: [**12**] = 44 and 14.5 μM. The same procedure was used for the tetraPIC data sets with [N_3_] = 38.6 μM and [**12**] = 38.6 and 12.7 μM.

## Supplementary Material



## Notes

Supporting Information reaction Schemes S1–S2; Supporting Information Tables S1–S2; Supporting Information Figures S1–S11: detailed reaction schemes; additional photophysical analysis data; MALDI-ToF spectra of **11**–**13** (PDF)
