# Supplementary material for: A Water-Soluble Fluorogenic Probe to Quantify Azide Concentrations in Biologically Relevant Environments
Source: Biomacromolecules. 2026 Jul 22;27(8):5139–49. doi: 10.1021/acs.biomac.6c00253 (PMC13463542; doi:10.1021/acs.biomac.6c00253)
Supplement: Supplementary file 1 [file bm6c00253_si_001.pdf]

Supplementary information for

A Water-Soluble Fluorogenic Probe to Quantify Azide Concentrations in Biologically Relevant Environments

Lotte Gerrits,[a,b] Lisa Verdellen,[a] Marie Peeters,[a] Arwen Gelderman,[a] Ulysse J. Gross,[a] Behrad Shaghaghi, [a,c] Roel Hammink,[c,d]* Paul H. J. Kouwer[a,b]*

[a] Dr. L. Gerrits, L. Verdellen, M. Peeters, A. Gelderman, U.J. Gross, B. Shaghaghi, Dr. P.H.J. Kouwer*
Institute for Molecules and Materials, Radboud University, Heyendaalseweg 135, 6525 AJ Nijmegen, The Netherlands.
E-mail: paul.kouwer@ru.nl

[b] Dr. L. Gerrits, Dr. P.H.J. Kouwer*
Institute for Chemical Immunology, Geert Grooteplein 28, 6525 GA, Nijmegen, The Netherlands.

[c] Dr. Behrad Shaghaghi, Dr. Roel Hammink*
Department of Medical BioSciences, Radboudumc, Geert Grooteplein 26, 6525 GA, Nijmegen, The Netherlands. E-mail: role.hammink@radboudumc.nl

[d] Dr. Roel Hammink*
Division of Immunotherapy, Oncode Institute, Radboudumc, 6525 GA, Nijmegen, The Netherlands.

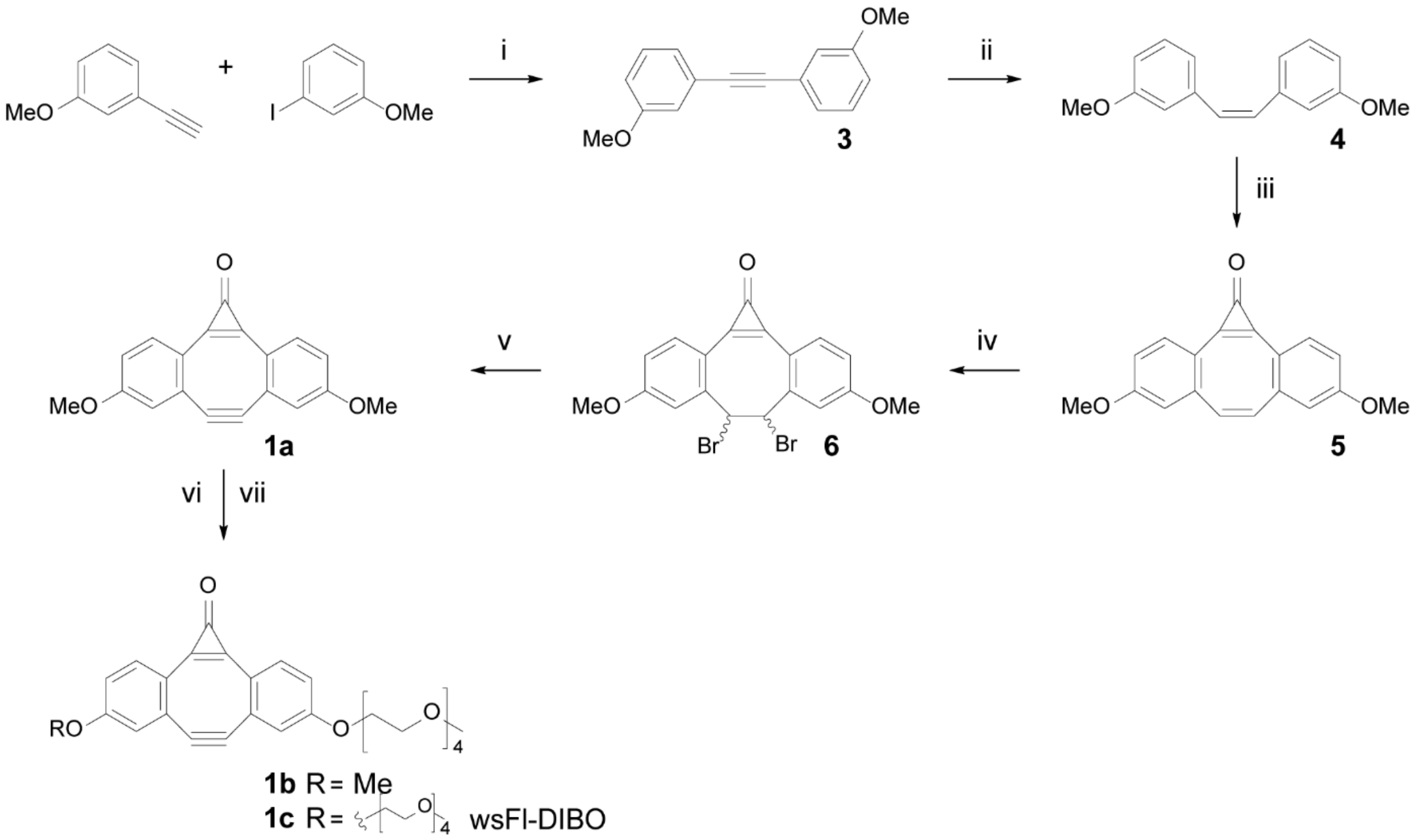

Scheme S1 Synthesis of wsFl-DIBO. i) $Pd(PPh_3)_4$, CuI, DIPEA, THF, reflux 18 h, 99%; ii) H_2, Lindlar's catalyst, quinoline, heptane, rt, 1h, 99%; iii) $AlCl_3$, tetrachlorocyclopropene, DCM, –78 °C to rt, 6 h, H_2O, 47%; iv) Br_2, DCM, 0 °C to rt, 4 h, 65%; v) KOH, EtOH, rt, 18 h, 61%; vi) BBr_3, DCM, –78 °C to rt, 72 h, no work-up; vii) Ts-TEG-OMe, K_2CO_3, DMF, 50 °C, 18 h, 7.5% (**1b**) and 20% (**1c**).

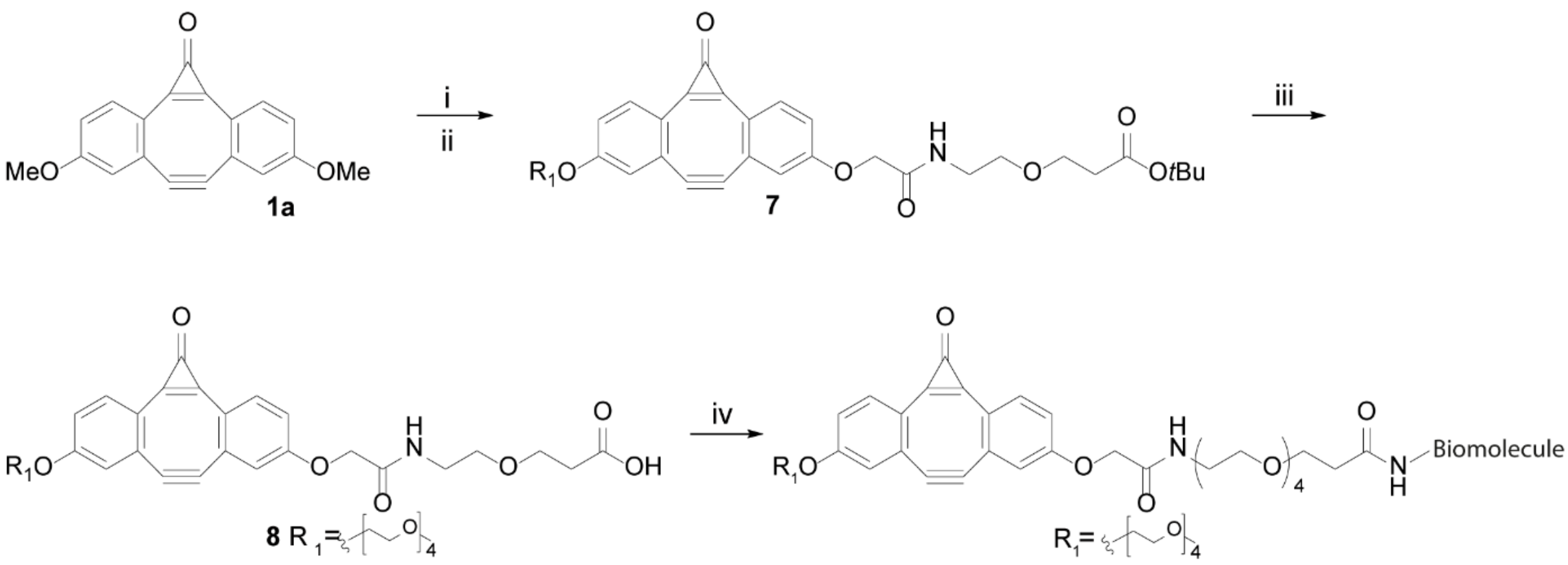

Scheme S2. Synthesis of bifunctional wsFl-DIBO. i) BBr_3, DCM, –78 °C to rt, 72 h, no work up; ii) 1. bromoacetamido-PEG_4-*t*-butyl ester, K_2CO_3, DMF, 45 °C, 72 h 2. Ts-PEG_4-OMe, K_2CO_3, DMF, 45 °C, 18 h, 11%; iii) HCl in dioxane (4M), DCM, 0 °C to rt, 92 h, 71%; iv) 1 N-hydroxysuccinimide, MES buffer, rt, 1 h, no work up; 2. GYIGSR/GRGDS/NH_2-PEG_3-biotin/BSA, Borate buffer 50 mM pH = 8.5, rt, 18 h, no work up.

Table S1. Photophysical properties of **1c** in PBS at 20 °C.

Compound	λ_{abs} (nm)	λ_{em} (nm)	ε_{abs} ($M^{-1}cm^{-1}$)	Stokes shift (cm^{-1})	Q_f[a]
**1c**	362	425	2298	4095	0.014

[a] Fluorescence quantum yield Q_f determined with quinine sulfate (1.0 M in H_2SO_4) as standard.

Table S2. Photophysical properties of bifunctional wsFl-DIBO (**8**) and bifunctional wsFl-DIBO triazole (**10**) in PBS at 20 °C.

**Compound**	λ_{abs} (nm)	λ_{em} (nm)	ε_{abs} ($M^{-1}cm^{-1}$)	**Stokes shift** (cm^{-1})	Q_f[a]
**8**	360	480	2860	6944	0.3122
**9**	362	525	3738	8577	0.0017

[a] Fluorescence quantum yield Q_f determined with quinine sulfate (1.0 M in H_2SO_4) as standard.

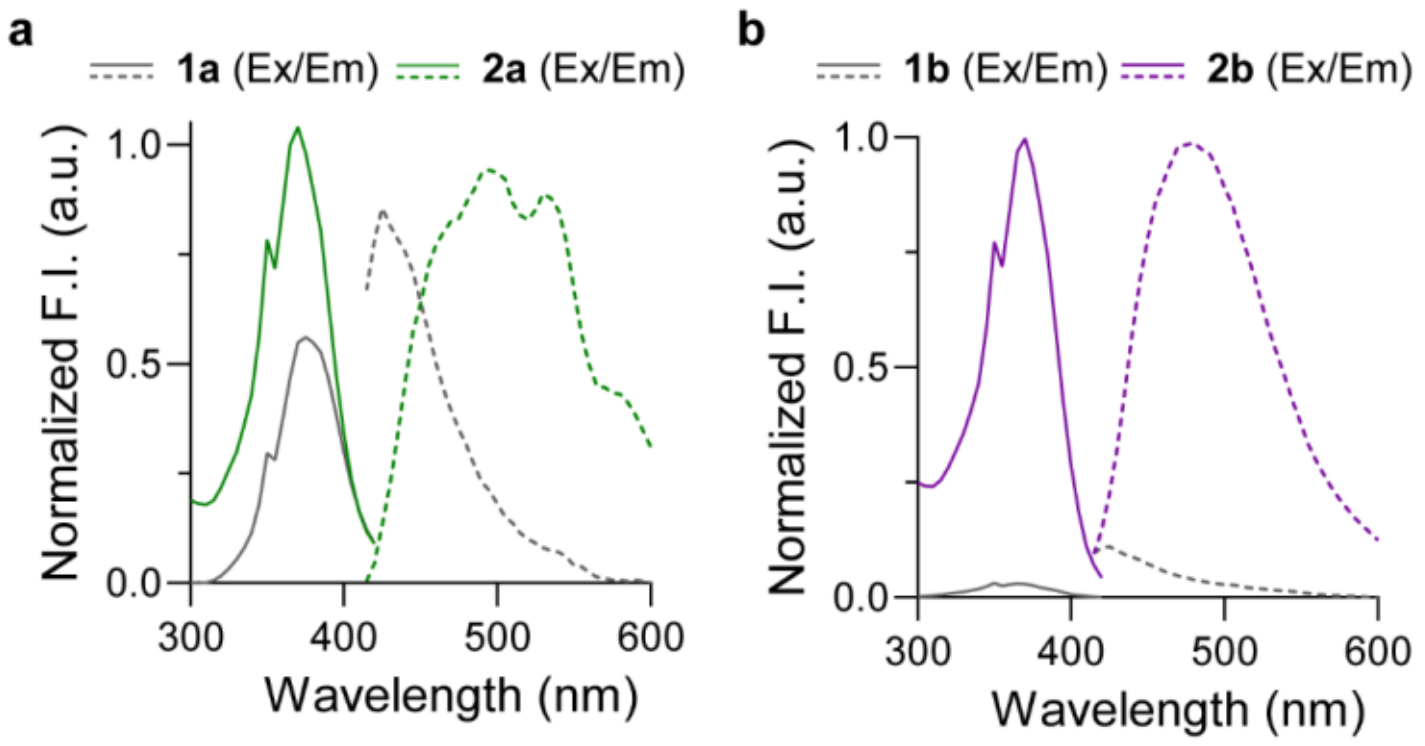

Figure S1. Fluorogenic properties of **1a** and **1b** and the corresponding triazole compounds **2a** and **2b**. a) excitation (solid lines) and emission (dashed lines) spectra of **1a** and corresponding triazole **2a** in PBS at 20 °C. b) excitation (solid lines) and emission (dashed lines) spectra of **1b** and corresponding **2b** in PBS at 20 °C.

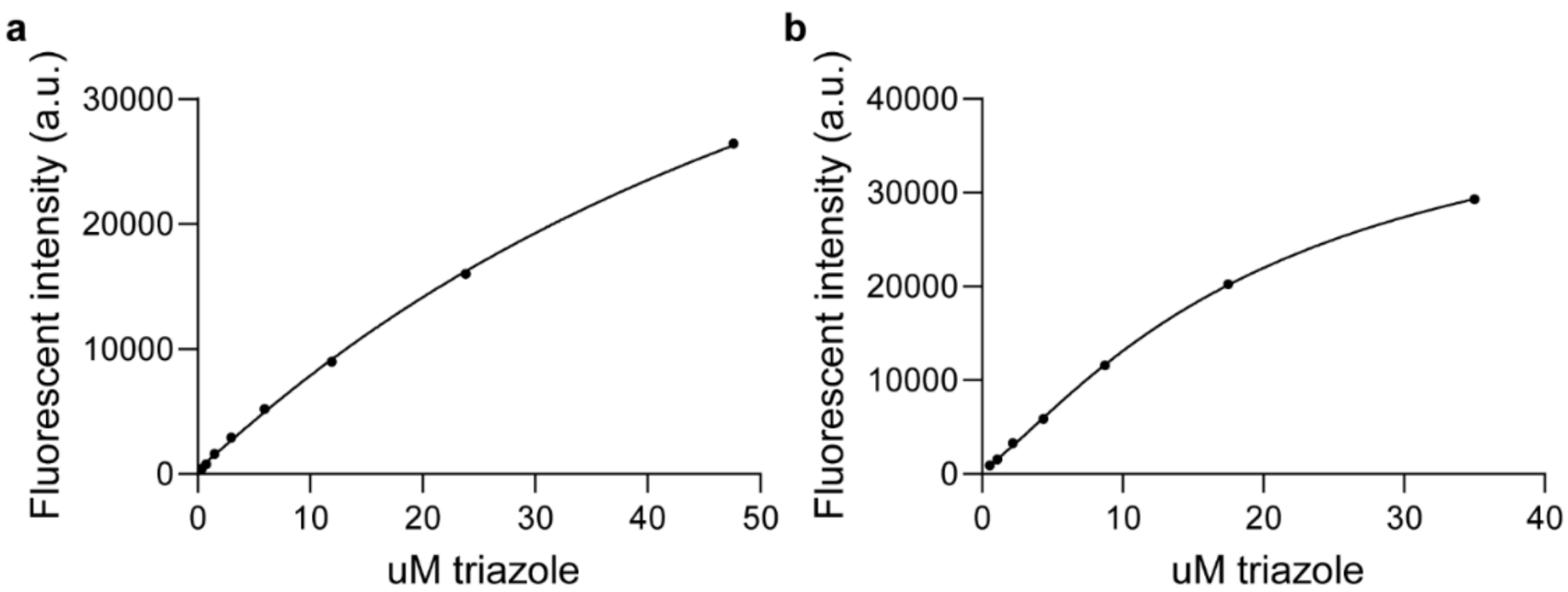

Figure S2. Calibration curves used for the quantification of available azides on tri- and tetraPIC scaffolds. wsFl-DIBO was incubated with known concentrations of azido-PEG$_3$-amine at 5 °C (a) or 20 °C (b), after which dilution series were made and the corresponding fluorescent intensity for each triazole concentration was measured. The obtained calibration curves were fitted with a 4-parameter logistic model and used to determine the triazole concentration on the respective tri- and tetraPIC scaffolds after incubation with wsFl-DIBO.

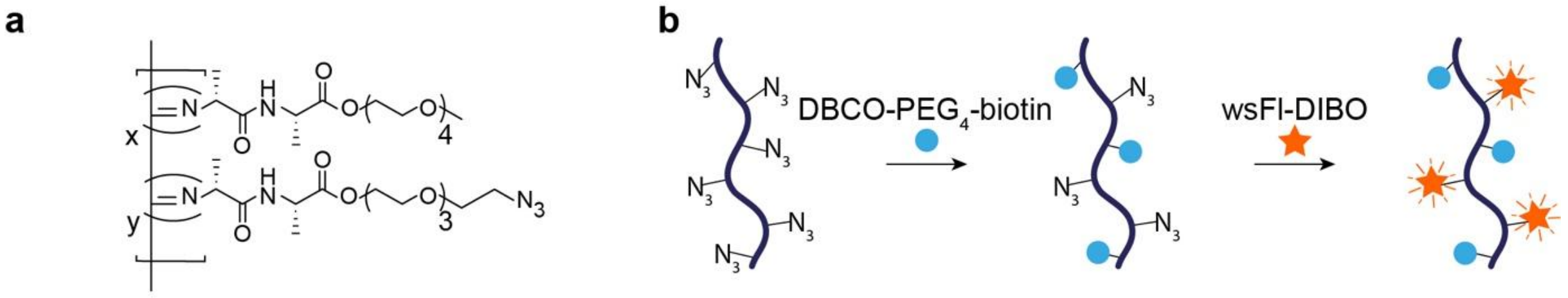

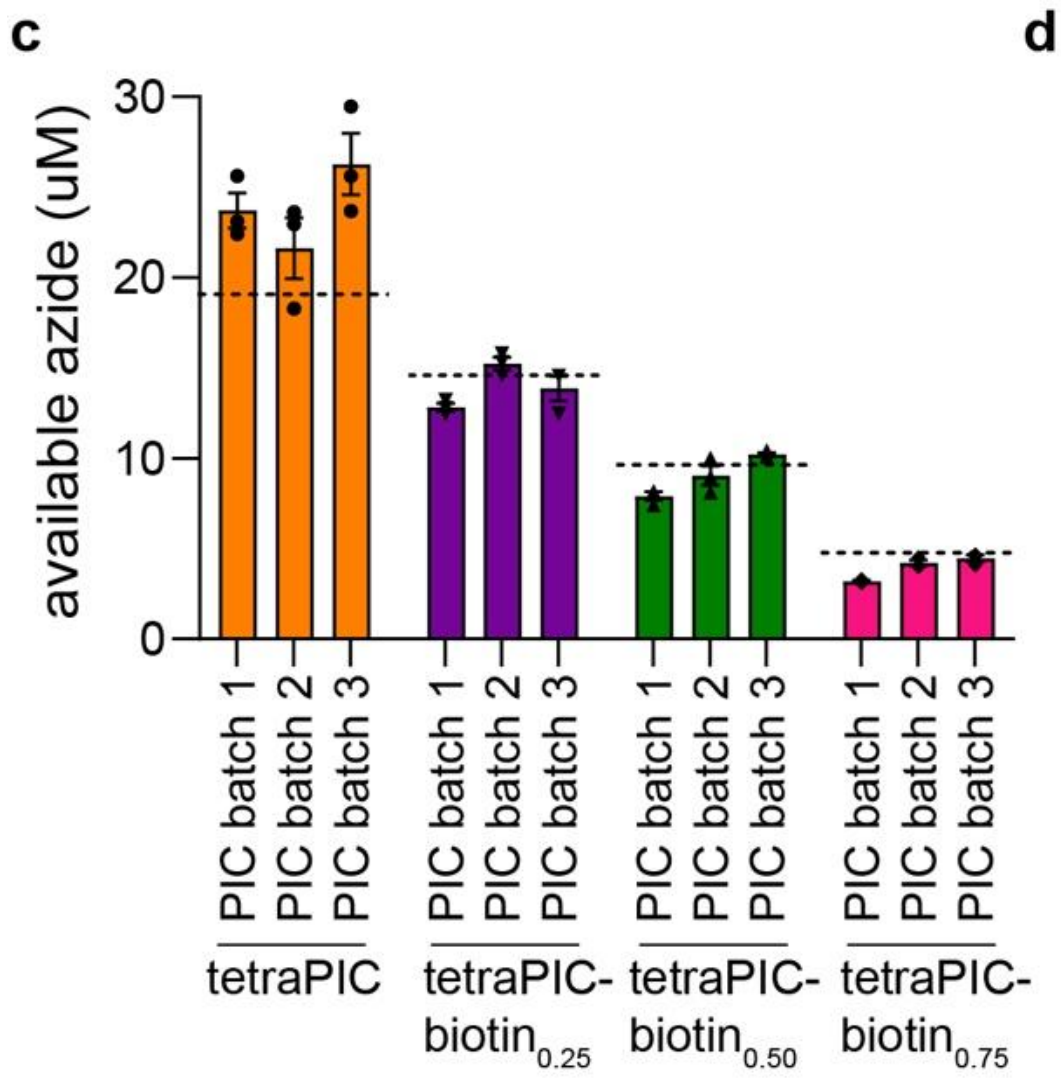

d

sample	average fraction N_3 monomer (y)
tetraPIC	0.041±0.004
tetraPIC-biotin$_{0.25}$	0.024±0.002
tetraPIC-biotin$_{0.50}$	0.016±0.002
tetraPIC-biotin$_{0.75}$	0.007±0.001

Figure S3. Azide quantification on multiple tetraPIC scaffolds. a) Chemical structure of tetraPIC scaffold. b) Schematic overview of biotin conjugation to tetraPIC and subsequent wsFl-DIBO conjugation for free azide concentration analysis on the scaffolds. c) Measured available azide on concentrations for tetraPIC scaffolds before and after biotin conjugation. Dashed lines indicate the expected available azide concentration assuming the fraction of azide functionalized monomer (y) on tetraPIC is 0.033 and assuming full consumption of DBCO-PEG_4-biotin. Data are represented as mean±SEM, N=3. d) Table showing average measured fraction of azide monomers calculated from the fractions of PIC batches 1, 2 and 3. For each batch, the azide monomer fraction was calculated from the concentration of available azide after addition of wsFl-DIBO (carried out in triplo). Data are represented as the mean±SD, N=3.

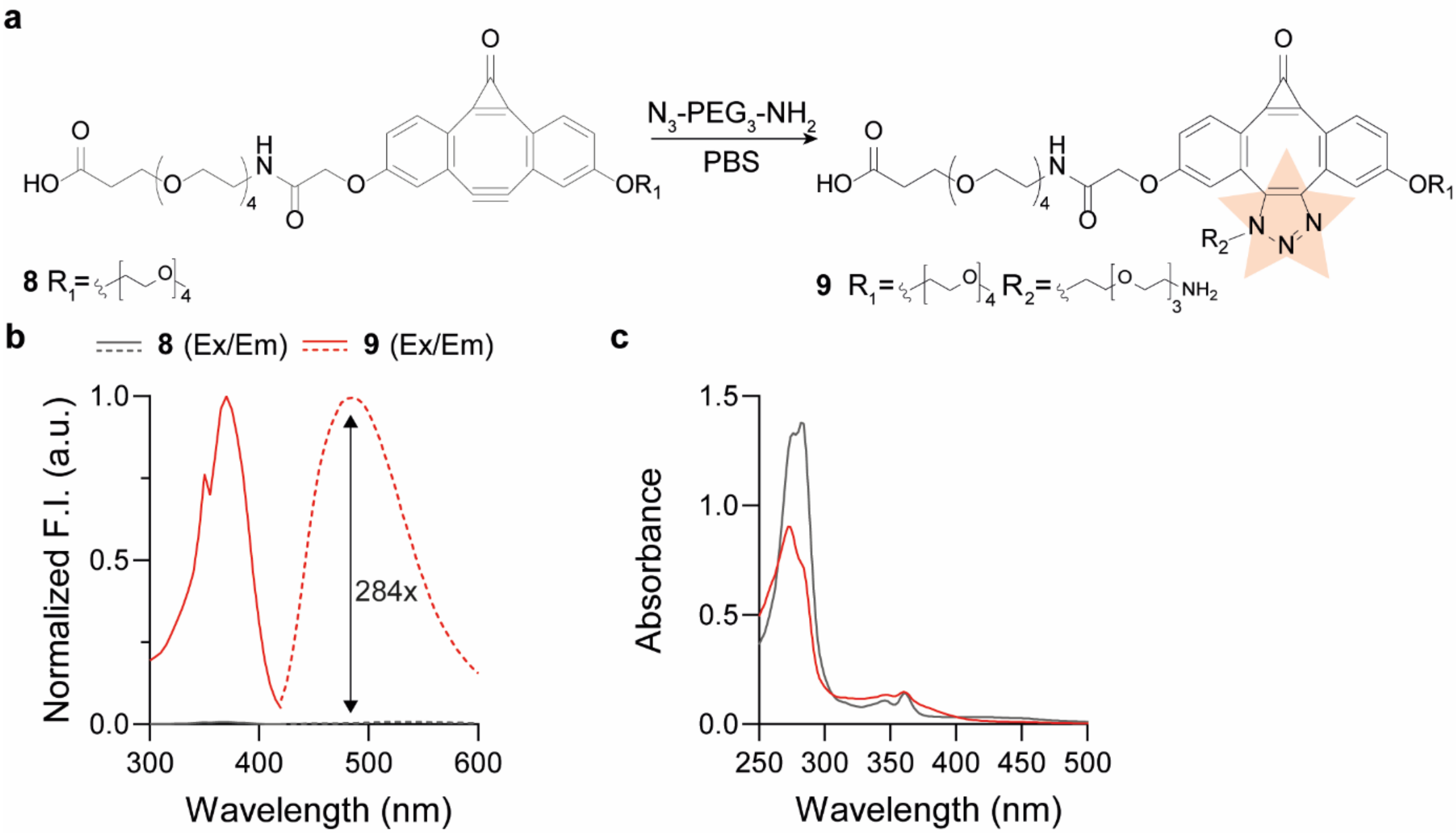

Figure S4. Fluorogenic COOH-wsFl-DIBO. a) SPAAC reaction between COOH-wsFl-DIBO (**8**) and azide-PEG3-amine to obtain the fluorescent triazole (**9**). b) excitation (solid lines) and emission (dashed lines) spectra of **8** and **9** in PBS at 20 °C. c) Absorption spectrum of **8** and **9** in PBS at 20 °C.

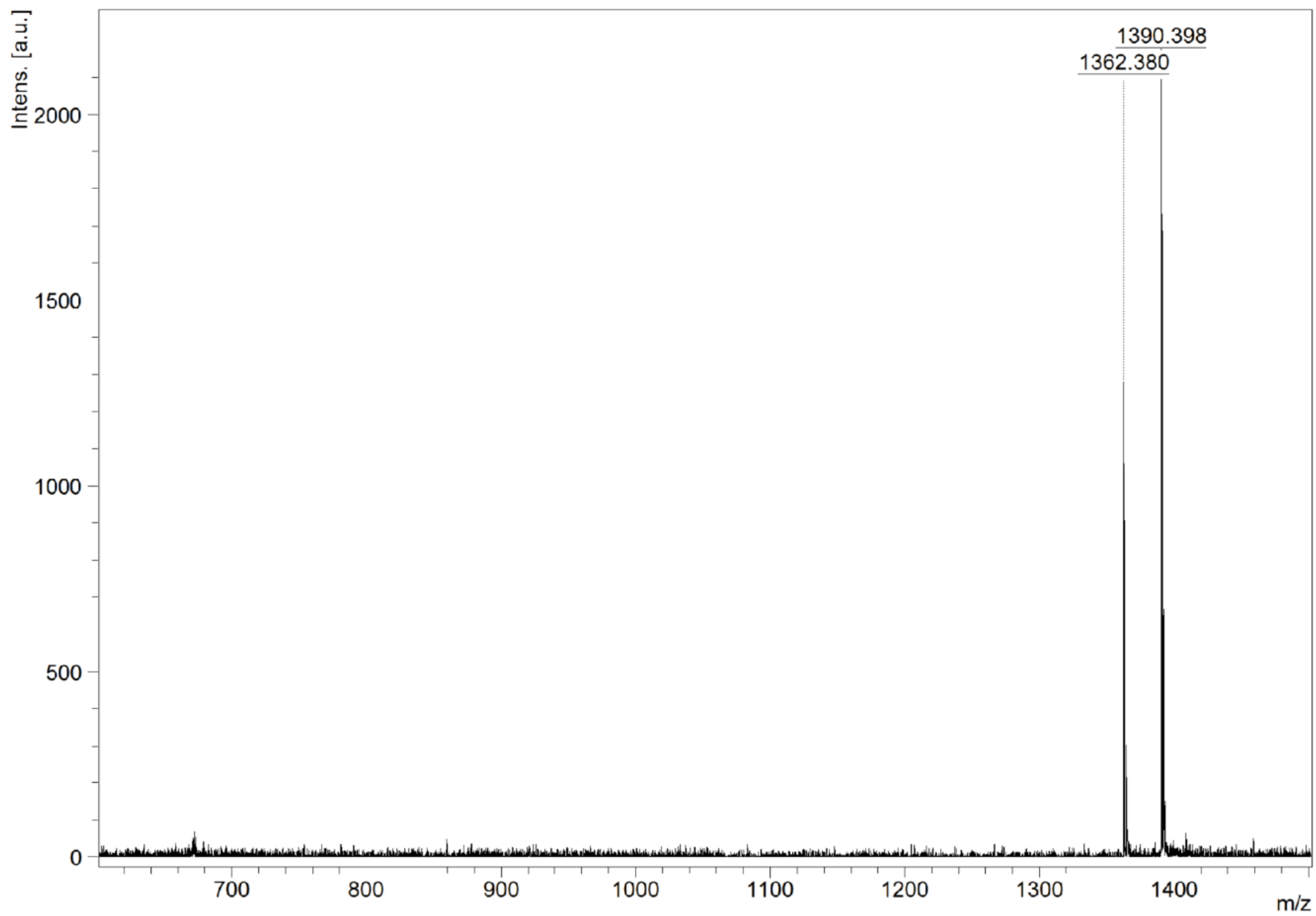

Figure S5. MALDI-TOF spectrum of YIGSR-wsFl-DIBO (**11**).

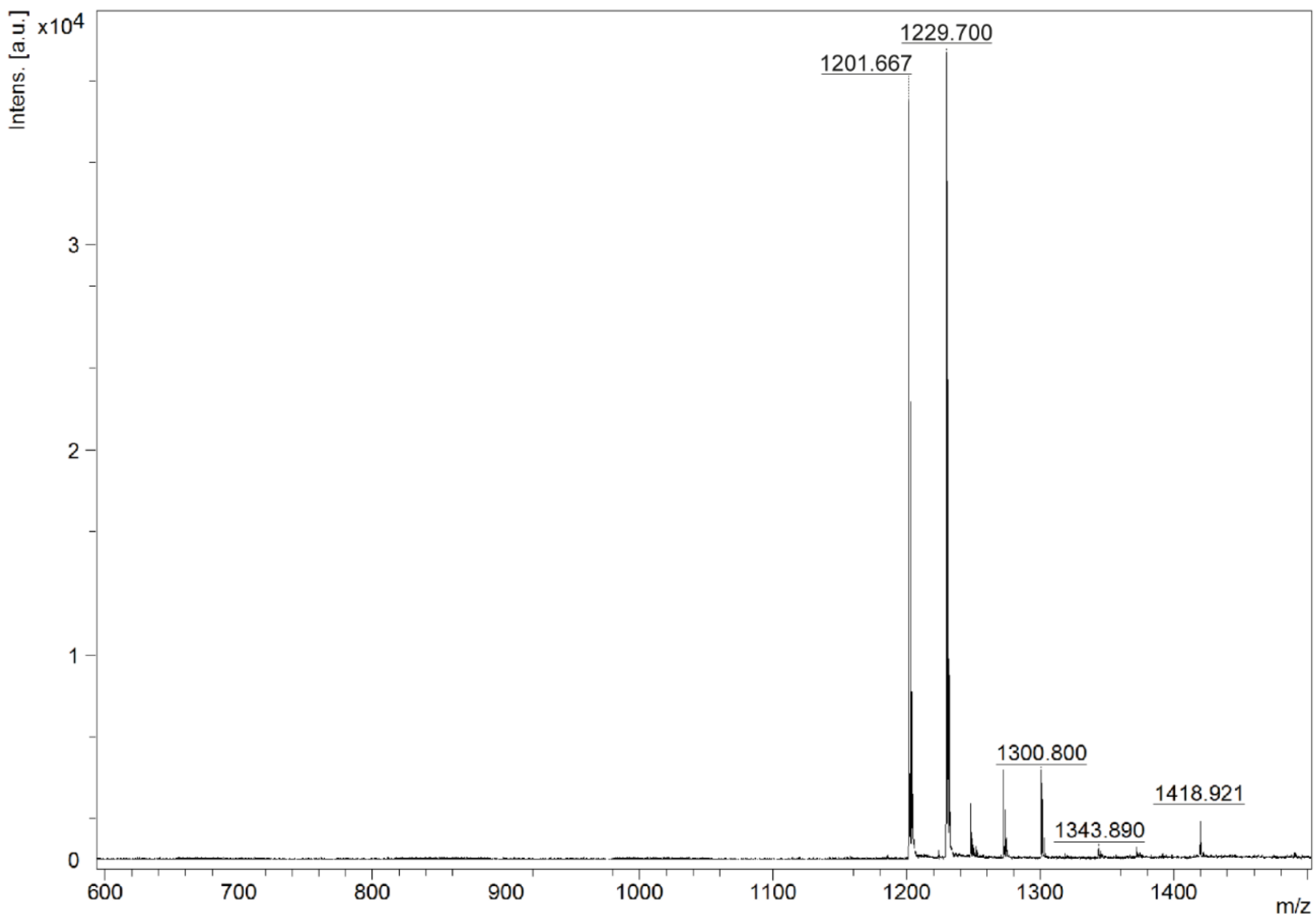

Figure S6. MALDI-TOF spectrum of GRGDS-wsFl-DIBO (**12**).

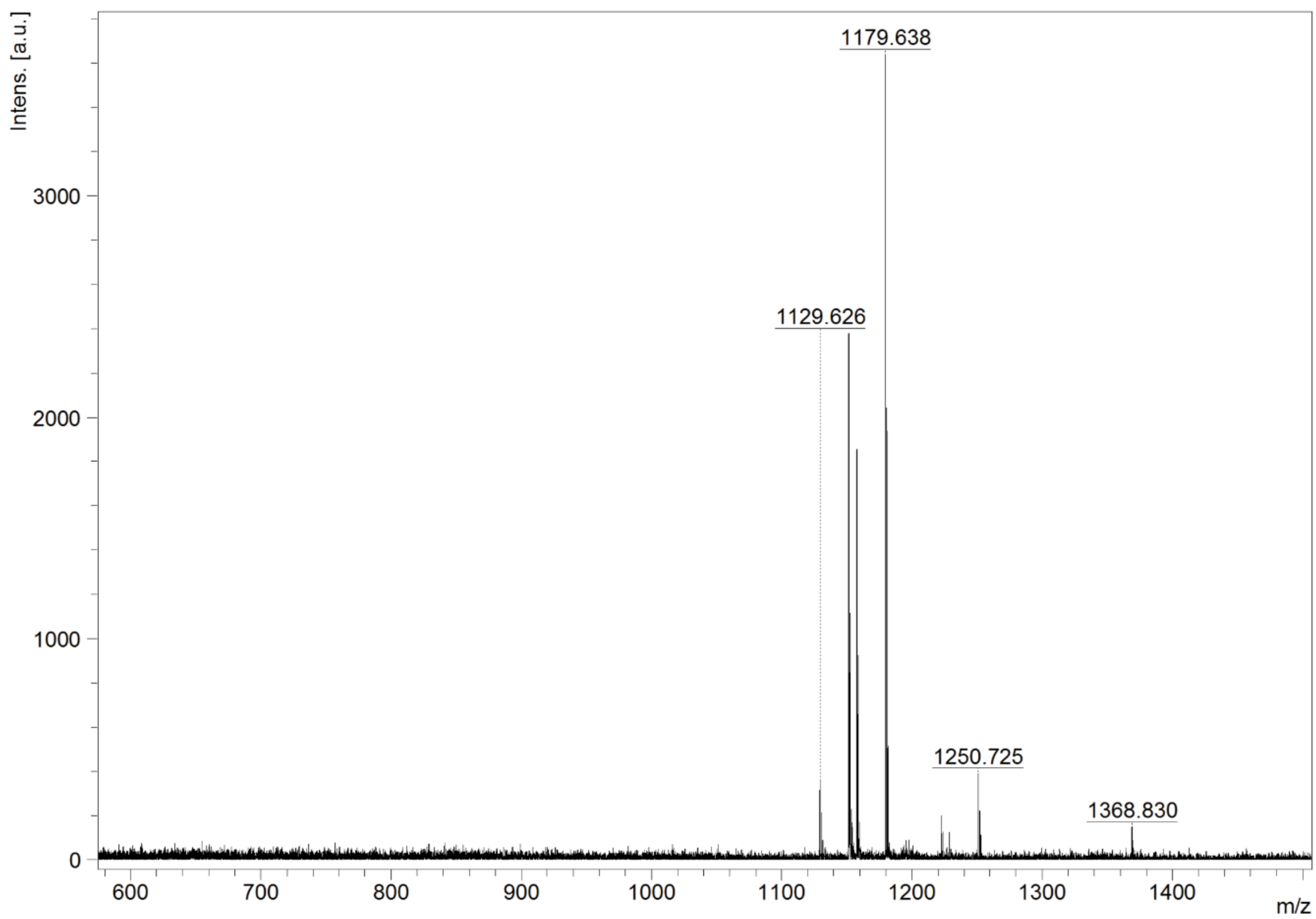

Figure S7. MALDI-TOF spectrum of Biotin-wsFl-DIBO (**13**).

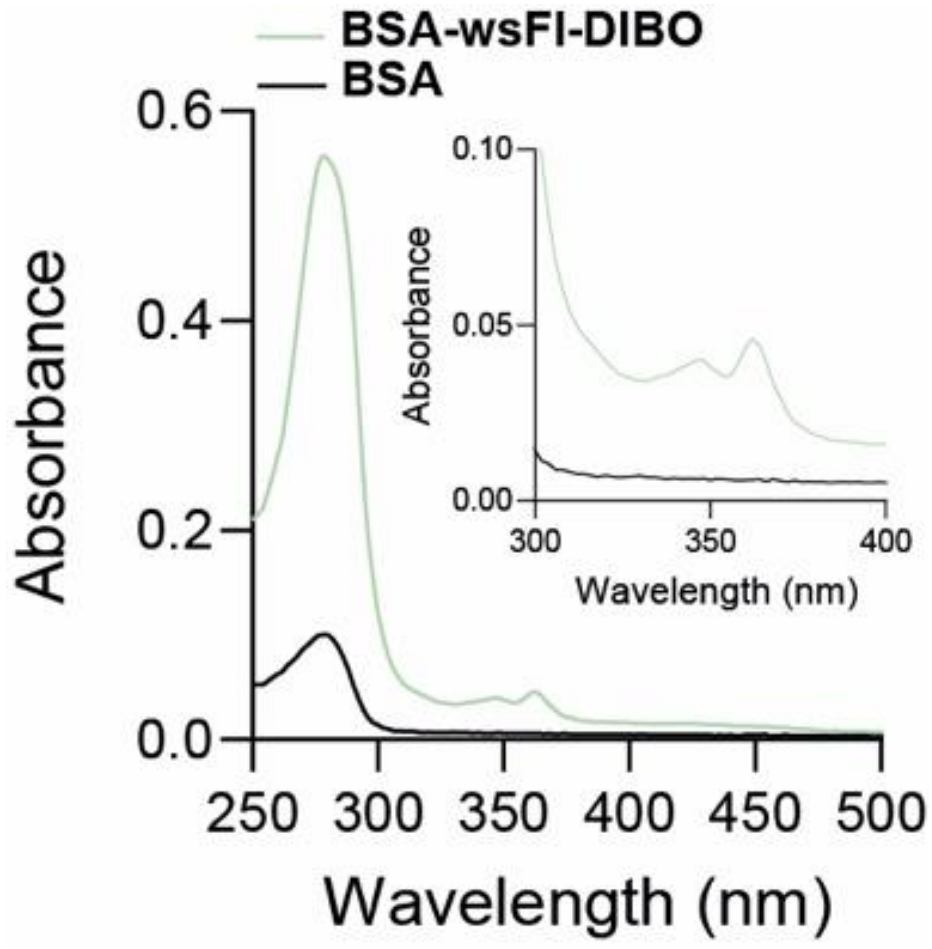

Figure S8. absorbance spectrum of BSA (black) and BSA-wsFl-DIBO (pale green) in PBS at 20 °C. The zoom-in of the absorbance spectrum of BSA and BSA-wsFl-DIBO, with absorbance peaks at 346 and 362 nm indicates successful conjugation of COOH-wsFl-DIBO.

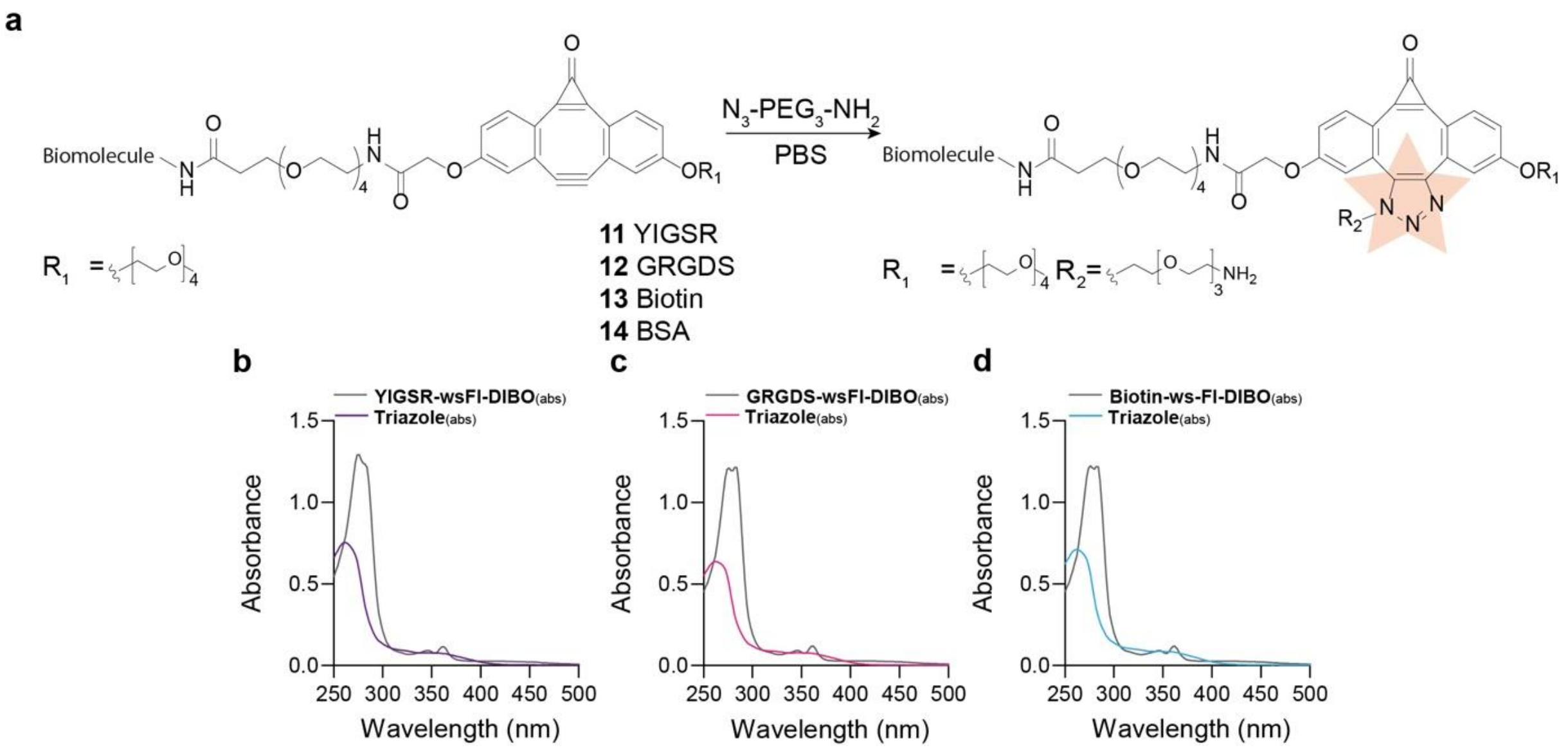

Figure S9 Fluorogenic biomolecule-wsFl-DIBO. a) SPAAC reaction between Biomolecule-wsFl-DIBO (**11**, **12**, **13,14**) and azide-PEG$_3$-amine to obtain the fluorescent triazole derivative. b) Absorption spectrum of YIGSR-wsFl-DIBO (**11**) and the corresponding triazole in PBS at 20 °C. c) Absorption spectrum of GRGDS-wsFl-DIBO (**12**) and the corresponding triazole in PBS at 20 °C. d) Absorption spectrum of Biotin-wsFl-DIBO (**13**) and the corresponding triazole in PBS at 20 °C.

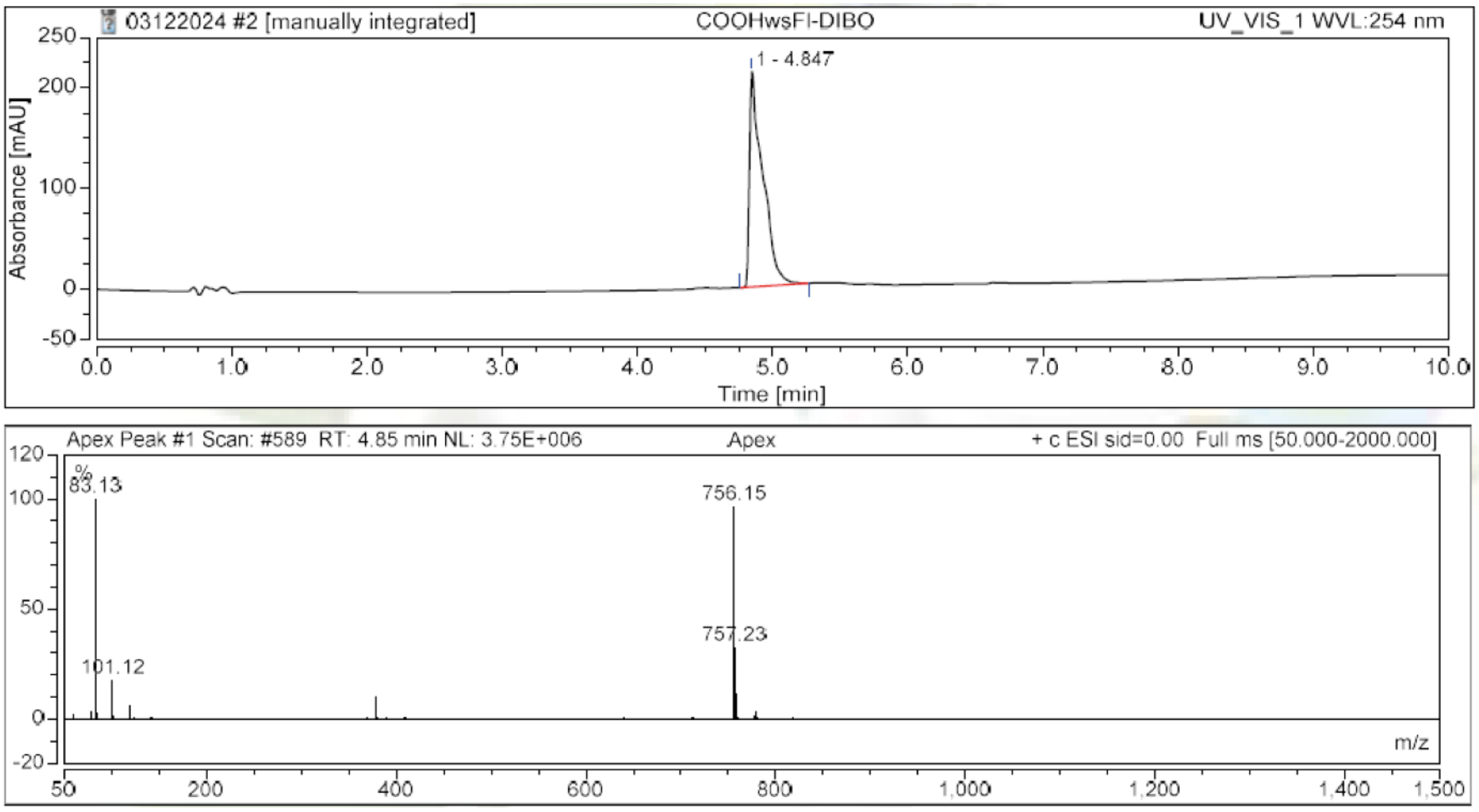

Figure S10. LCMS spectrum of COOH-wsFl-DIBO (**8**). (m/z): $[M+H]^+$ calcd. for $C_{39}H_{49}NO_{14}$, 756.82; found, 756.02

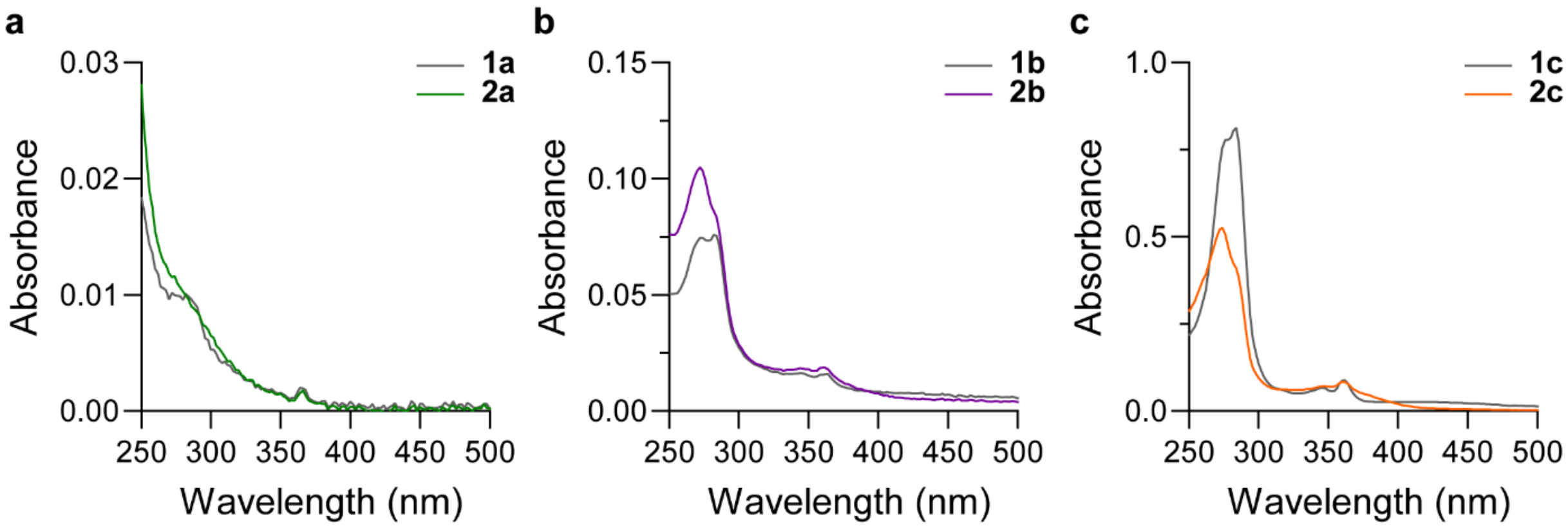

Figure S11. a) Absorption spectrum of **1a** and corresponding triazole **2a** in PBS at 20 °C. b) Absorption spectrum of **1b** and corresponding triazole **2b** in PBS at 20 °C. c) Absorption spectrum of **1c** and corresponding triazole **2c** in PBS at 20 °C.

References

[12] F. Friscourt, C. J. Fahrni, G.-J. Boons, *J. Am. Chem. Soc.* **2012**, *134*, 18809-18815.

[32] K. Liu, S. M. Mihaila, A. Rowan, E. Oosterwijk, P. H. J. Kouwer, *Biomacromolecules* **2019**, *20*, 826-834.

[46] D. W. Paley, D. F. Sedbrook, J. Decatur, F. R. Fischer, M. L. Steigerwald, C. Nuckolls, *Angew. Chem. Int. Ed.* **2013**, *52*, 4591-4594.

[47] P. Sun, G. Yan, Q. Tang, Y. Chen, K. Zhang, *Polymer* **2015**, *64*, 202-209.

[48] G. A. Crosby, J. N. Demas, *J. Phys. Chem.* **1971**, *75*, 991-1024.

[49] S. Mandal, Z. H. Eksteen-Akeroyd, M. J. Jacobs, R. Hammink, M. Koepf, A. J. Lambeck, J. C. van Hest, C. J. Wilson, K. Blank, C. G. Figdor, *Chem. Sci.* **2013**, *4*, 4168-4174.
